# Mapping the Cerebral Organoid Landscape: A Systematic Review of Preclinical 3D Models in Neuroscience

**DOI:** 10.1002/adhm.202504889

**Published:** 2026-03-11

**Authors:** Anna Wolfram, Vanessa Arnold, Maik Wolfram‐Schauerte, Anastassiya Moskalchuk, Caroline Trust, Marie Vontz, Mona Scheurenbrand, Vijayasarathy Sampath‐Kumar, Sogand Ahari, Carlos Romero‐Nieto, Lisa Sevenich

**Affiliations:** ^1^ Department of Neurology and Interdisciplinary Neurooncology Hertie Institute for Clinical Brain Research University Hospital Tübingen Tübingen Germany; ^2^ M3 Research Center For Malignome Metabolome and Microbiome University Hospital Tübingen Tübingen Germany; ^3^ Graduate Training Centre of Neuroscience University of Tübingen Tübingen Germany; ^4^ Faculty of Pharmacy University of Castilla‐La Mancha Albacete Spain; ^5^ Instituto Regional de Investigación Científica Aplicada (IRICA) University of Castilla‐La Mancha Ciudad Real Spain; ^6^ Institute for Bioinformatics and Medical Informatics Department of Computer Science University of Tübingen Tübingen Germany; ^7^ Cluster of Excellence iFIT (EXC 2180) 'Image‐Guided and Functionally Instructed Tumor Therapies' University of Tübingen Tübingen Germany

**Keywords:** CNS, ex vivo models, neurodevelopment, neurodegeneration, neurooncology, organoid database

## Abstract

Cerebral organoids are complex, three‐dimensional (3D) dynamic models that recapitulate key features of brain development and disease. These systems serve as bioengineerable platforms with diverse architectures and customizable properties, enabling advances in both basic and translational neuroscience. Despite rapid adoption across neurodevelopment, neurodegeneration, and neuro‐oncology, the field remains fragmented, with substantial methodological variability and no standardized framework for model selection. A systematic review of 738 original studies published between 2014 and 2024, drawn from 3631 articles across PubMed, Semantic Scholar, and OpenAlex, reveals that human induced pluripotent stem cell‐derived cerebral organoids and neurodevelopmental studies dominate the field. In contrast, applications in neurodegeneration, brain metastases, and non‐human systems remain limited, narrowing the translational scope. To address the challenge of navigating this expanding literature, *OrganoidMap* is introduced—an open‐access, interactive web platform for exploring, filtering, and comparing cerebral organoid models across disease areas, cell sources, and methodological features. *OrganoidMap* enables the identification of appropriate models for specific experimental goals and reveals underexplored research areas. This synthesis establishes a scalable foundation for enhancing transparency, reproducibility, and model selection in organoid‐based research, setting a new benchmark for how neuroscience and biomaterials communities organize, share, and advance cerebral organoid science.

## Graphical abstract

Cerebral organoids are transforming brain research, yet the field remains fragmented. This comprehensive systematic review maps 738 studies published between 2014 and 2024 to uncover trends, gaps, and opportunities across neuroscience. Introducing *OrganoidMap*—an interactive, open‐access platform to explore and compare models—this work enables researchers to navigate complexity, improve reproducibility, and accelerate discovery in neurodevelopment, neurodegeneration, and neuro‐oncology.

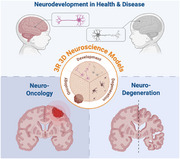

## 1 Introduction

The mammalian brain is a highly complex biological system, characterized by intricate cytoarchitecture, cellular diversity, and region‐specific developmental programs [1, 2, 3]. These features make experimental modeling particularly challenging, especially when seeking to capture species‐specific neurobiological traits [4, 5]. Traditionally, in vivo models have relied heavily on rodents due to their accessibility and well‐established protocols [6, 7]; however, these models often fail to replicate features unique to higher‐order species, particularly primates and humans [8]. Modeling human brain development introduces additional barriers, including limited tissue access and strict ethical regulations [9, 10, 11], underscoring the need for species‐relevant systems that are not only biologically meaningful but also experimentally tractable. Moreover, ethical principles such as the 3Rs (Replacement, Reduction, Refinement), as outlined in EU Directive 2010/63 [12], emphasize the importance of minimizing animal use by promoting alternatives and improving experimental design [12, 13, 14, 15, 16]. Recent advances in stem cell biology have led to the emergence of three‐dimensional (3D) brain organoids [17, 18, 19, 20, 21, 22] —self‐organizing tissue constructs that recapitulate key aspects of in vivo brain development, including neurogenesis, regional patterning, cellular differentiation, and rudimentary tissue organization [23, 24, 25]. Within this category, cerebral organoids are particularly notable, as they model forebrain and cortical regions relevant to higher‐order cognition and neuropsychiatric and neurodegenerative disorders [26, 27]. While often used interchangeably with “brain organoids”, cerebral organoids exhibit more defined anatomical and developmental identities than general brain organoid models [28].

Importantly, cerebral organoids represent not only biological models but also highly complex, dynamic, and intermodal living systems [29] with heterogeneous architectures, emergent behaviors, and tunable experimental parameters [30, 31]. These properties position them at the intersection of neuroscience, biomaterials science, and translational medicine, offering unprecedented opportunities to explore fundamental mechanisms and develop clinically relevant applications [31, 32]. Cerebral organoids have been developed from various mammalian species and cell sources, each with distinct advantages and limitations [27, 33, 34, 35, 36, 37]. In rodents, organoids are typically derived from neural stem or progenitor cells isolated from neurogenic regions [38, 39]. In primates and humans, however, ethical and technical constraints favor the use of pluripotent stem cells, such as embryonic stem cells (ESCs) [40, 41, 42, 43] or, increasingly, induced pluripotent stem cells (iPSCs) [44, 45, 46, 47, 48]. While many studies employ standardized iPSC lines, an expanding body of work uses patient‐derived iPSCs to generate personalized cerebral organoids [49], effectively creating “patient avatars” [50, 51] that capture genetic individuality and enable precision modeling of disease susceptibility, progression, and therapeutic response [52, 53]. Unlike traditional two‐dimensional cultures, these three‐dimensional systems better reflect the native brain microenvironment, providing modular, scalable, and customizable platforms for basic research, drug discovery, and personalized medicine [54, 55, 56].

**FIGURE 1:**
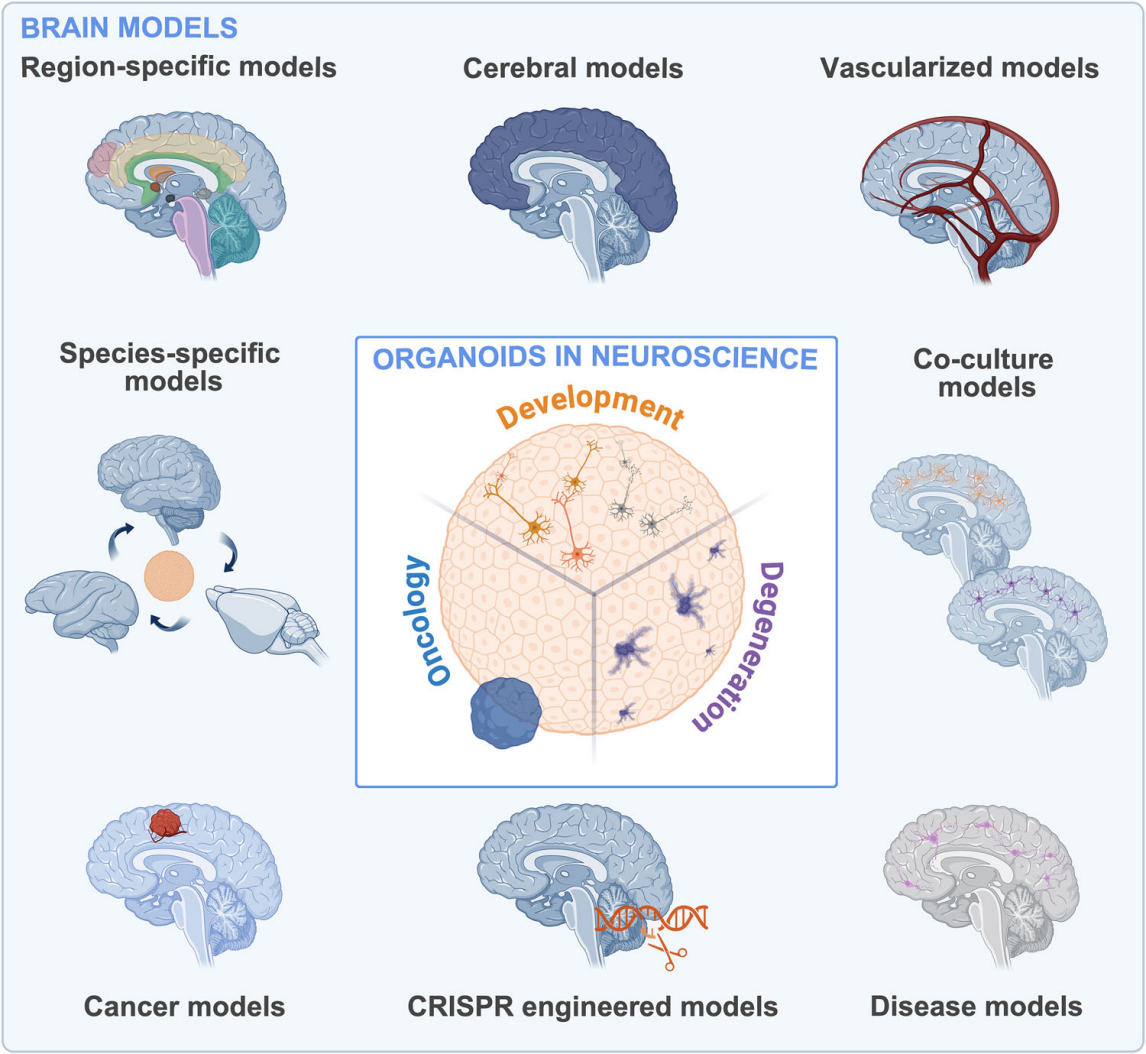
Major domains of cerebral organoid research in neuroscience. Cerebral organoids are applied across the three major neuroscience domains—neurodevelopment, neurodegeneration, and neuro‐oncology—as models of specific brain regions, cerebral identity, vascularization, co‐cultures with supporting cell types such as microglia and astrocytes, disease processes, CRISPR‐based genetic engineering, cancer biology, and species‐specific differences. Created with BioRender.com.

In just over a decade, the cerebral organoid field has expanded rapidly, yielding a diverse array of models, methods, and applications [57], as schematically illustrated in Figure 1. This remarkable growth, however, has resulted in a fragmented landscape, with substantial methodological heterogeneity and no unified framework to guide researchers in model selection, experimental design, or identification of underexplored research areas. While several prior reviews have addressed specific applications or subtypes of cerebral organoids, a comprehensive, systematic synthesis across neuroscience domains remains lacking. Here, we present the first comprehensive systematic review of mammalian cerebral and brain‐region‐specific organoid models applied across three major domains of neuroscience: neurodevelopment [11, 58, 59] (e.g., brain patterning [60, 61, 62], neurogenesis [61, 63, 64]); neurodegeneration [65] (e.g., Alzheimer's disease (AD) [66], Parkinson's disease (PD) [67] and amyotrophic lateral sclerosis (ALS) [68]) and neuro‐oncology [69] (e.g., glioblastoma (GBM) [70, 71, 72] and brain metastases [73, 74, 75]).

Drawing on 738 original studies published between 2014 and 2024 and sourced from 3631 articles across PubMed, Semantic Scholar, and OpenAlex, we classify models by species, cell source, neuroscience focus, and experimental application, highlighting their respective strengths and limitations. In addition, we introduce *OrganoidMap* (https://organoidmap.cs.uni‐tuebingen.de/), an open‐access, interactive web platform designed to help researchers explore, filter, and compare cerebral organoid models across key parameters based on our extensive, systematic literature research. Together, this systematic review and its companion platform uniquely integrate a multi‐domain synthesis with open‐access digital infrastructure, providing not only a practical guide for informed model selection, but also a scalable framework for field‐wide comparison and future benchmarking. *OrganoidMap* exemplifies open‐source scientific communication, offering a blueprint for how dynamic, user‐centered tools can enhance transparency, reproducibility, and cross‐disciplinary collaboration. This combined resource provides a foundational resource for advancing cerebral organoid research as a dynamic interface between neuroscience, biomaterials science, and translational innovation, with broad implications for future integration into biofabrication, organoid‐on‐chip systems, and personalized therapeutic pipelines.

## 2 Results

**FIGURE 2:**
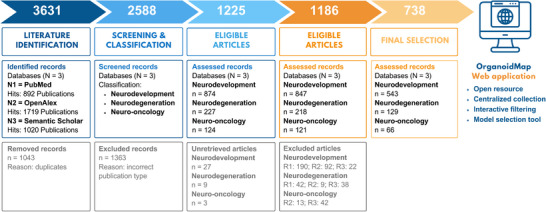
PRISMA 2020 flow diagram of study selection. Records identified: 3631; after duplicates removed: 2588; full‐text articles assessed: 1225; studies included: 738. Exclusion reasons: R1 – Incorrect model, R2 – Off‐topic, R3 – Incorrect article type. Final selection formed the basis for data available via the interactive web application *OrganoidMap*. Adapted from Page et al. (2021), PRISMA 2020 flow diagram, licensed under CC BY 4.0 [76]. Data extraction: 01 Jan 2014‐09 Oct 2024. Created with Canva (https://www.canva.com).

The results from our search strategy are summarized in Figure 2. An initial query for cerebral organoid models across PubMed, OpenAlex, and Semantic Scholar identified 3631 records. After removing duplicates, 2588 unique articles remained. Title and abstract screening based on the keywords “neurodevelopment”, “neurodegeneration”, and “neuro‐oncology” yielded 1225 articles for full‐text review. Following full‐text assessment, 738 studies (28.5%; 738/2588) met the inclusion criteria and were retained for analysis (Supplementary Excel File: *OrganoidMap.xlsx*). Articles were excluded based on the following predefined criteria: R1, incorrect model (i.e., studies not employing brain organoids or related 3D neural models); R2, off‐topic (i.e., studies outside the scope of neurodevelopment, neurodegeneration, or neuro‐oncology); and R3, incorrect article type (e.g., reviews, commentaries, protocols, or conference abstracts).

**FIGURE 3:**
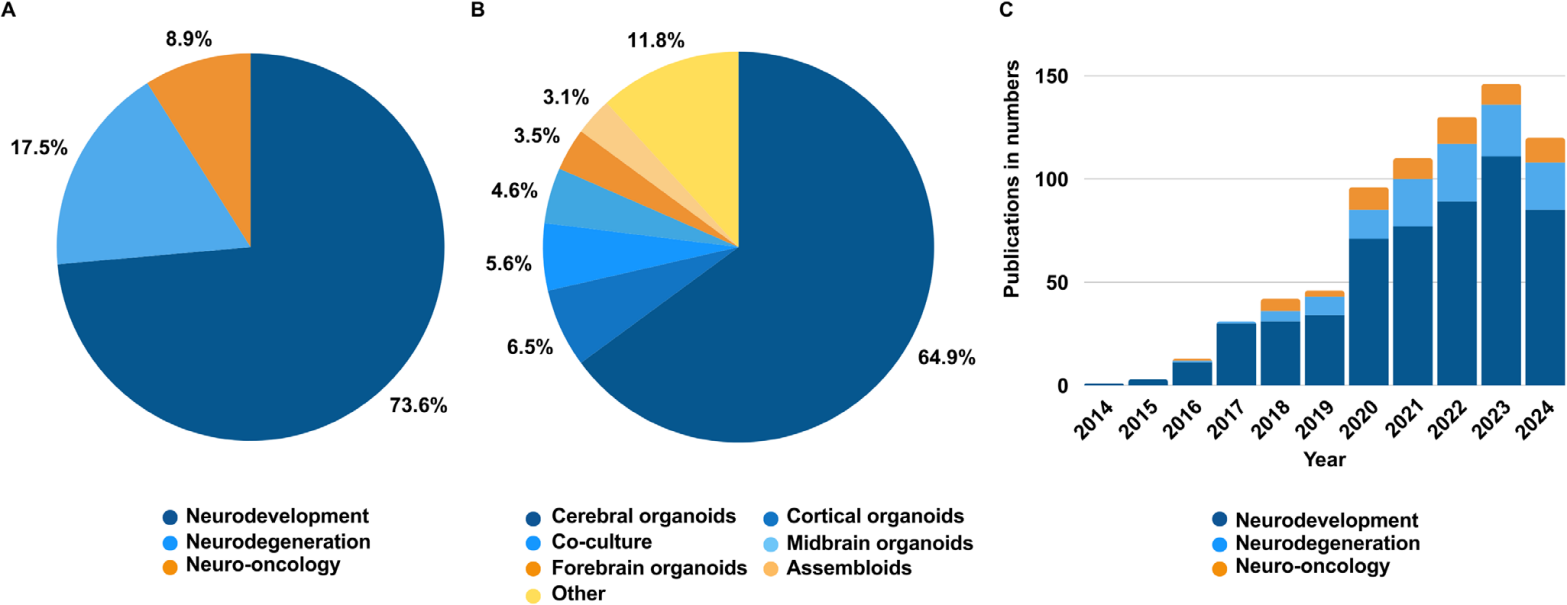
Distribution of included studies by domain, organoid type, and publication year. (A) Studies by domain: neurodevelopment (73.6%; 543/738), neurodegeneration (17.5%; 129/738), and neuro‐oncology (8.9%; 66/738). (B) Organoid models: cerebral (64.9%; 479/738), cortical, co‐culture, midbrain, forebrain, assembloids, and others. (C) Annual publication trends grouped by domain. Created with Canva (https://www.canva.com).

Among the included studies, brain organoids were most frequently applied in the context of neurodevelopment (73.6%; 543/738), followed by neurodegeneration (17.5%; 129/738) and neuro‐oncology (8.9%; 66/738) (Figure 3A). Regarding model type, cerebral organoids were used in 64.9% (479/738) of studies, followed by cortical organoids (6.5%; 48/738), co‐culture systems (5.6%; 41/738), midbrain organoids (4.6%; 34/738), forebrain organoids (3.5%; 26/738) and assembloids (3.1%; 23/738). An additional 11.8% (87/738) of studies employed other brain organoid models, including hindbrain organoids and regionally specified constructs (Figure 3B).

The annual number of publications has increased substantially over the past decade, with a marked acceleration beginning in 2020 (Figure 3C). (Note: Data were collected up to October 2024, which may explain the higher number of published articles observed in 2023). Thus, in 2023, 19.8% (146/738) of all included studies were published, reflecting sustained growth across all three domains (Figure 3C). Analysis of this figure further reveals that neurodevelopmental applications have consistently dominated the field since 2020. In contrast, although organoid use in neurodegeneration and neuro‐oncology is steadily increasing, these areas remain comparatively underrepresented. This distribution underscores both the current focus of brain organoid research and the potential for broader application in modeling diverse brain disease states. To support deeper exploration of these trends, we developed *OrganoidMap*—an interactive, open‐access web platform that enables users to filter, visualize, and compare the full dataset of 738 curated studies by research domain, disease model, cell source, and methodological features. The platform includes dynamic visualizations and search functions to assist in model selection, benchmarking, and experimental planning. *OrganoidMap* is freely accessible at https://organoidmap.cs.uni‐tuebingen.de.

The following sections provide a domain‐specific overview of organoid applications in neurodevelopment, neurodegeneration, and neuro‐oncology, with emphasis on condition modeling, translational applications, key advantages, and current limitations.

### 2.1 Cerebral Organoids in Neurodevelopment

Neurodevelopment is a tightly regulated process involving the specification, expansion, and maturation of neural cell types into regionally organized and functionally integrated brain circuits [77, 78]. Traditional models, including 2D cultures and rodent systems, capture only limited aspects of higher mammalian neurodevelopment and often fail to reflect the cellular diversity, developmental timing and species‐specific features of the brain [79]. Cerebral organoids have therefore emerged as a valuable platform to model early stages of brain development *ex vivo* [11, 80, 81, 82, 83].

In this systematic review, neurodevelopment represented the primary focus of organoid‐based neuroscience research, accounting for 543 of 738 studies (74%). Notably, 95.4% (518/543) of these studies used a human cellular background, primarily employing hiPSCs and/or hESCs, underscoring a clear emphasis on modeling human‐specific neurodevelopment.

**FIGURE 4:**
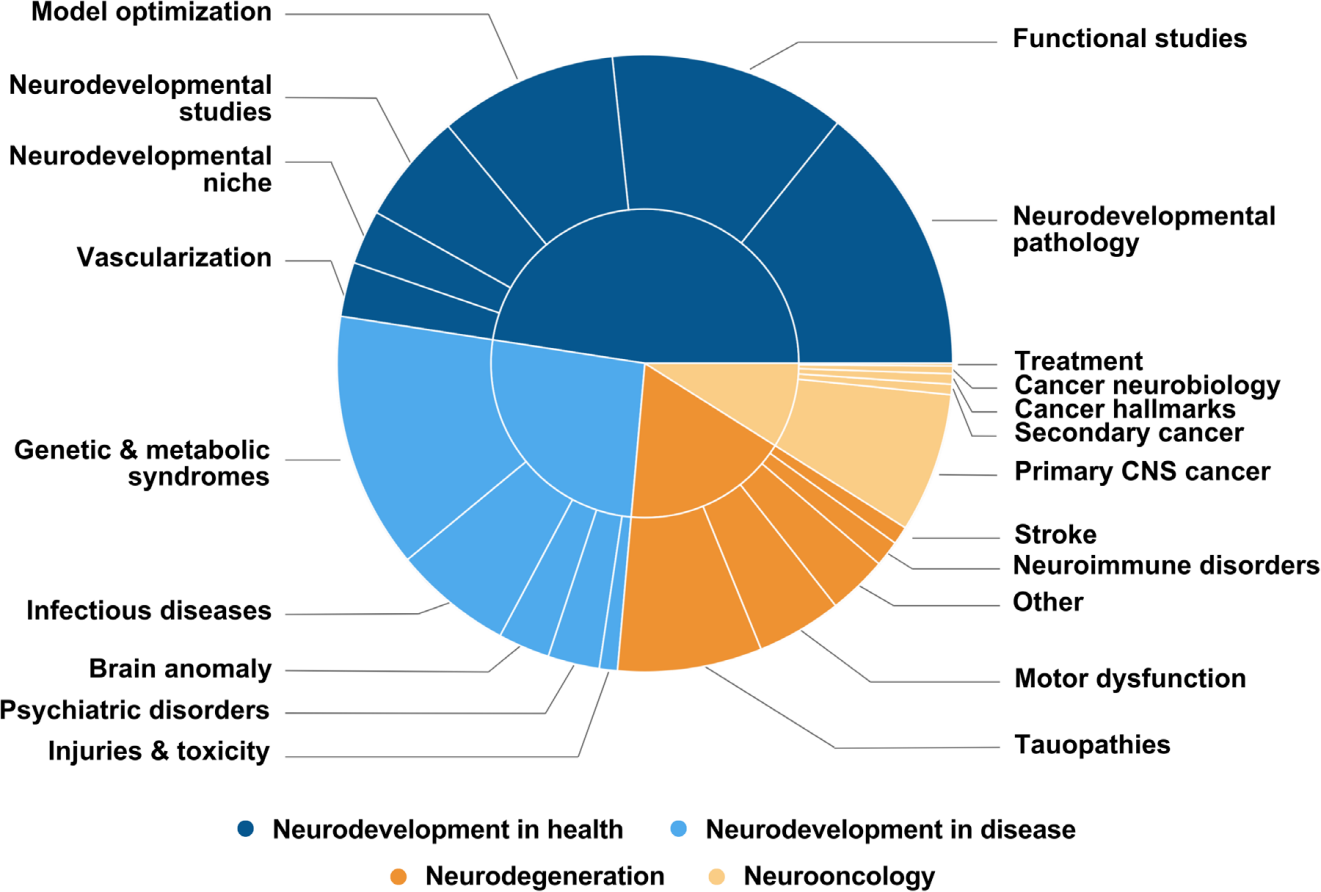
Organoids as models of neuroscience. Sunburst plot visualizing the distribution of 738 cerebral organoid studies across neurodevelopment (73.6%, 543/738), neurodegeneration (17.5%, 129/738) and neuro‐oncology (8.9%, 66/738). Neurodevelopmental studies were further subdivided into models of neurodevelopment in health (65.0%, 351/543) and disease (35.0%, 192/543). Thematic breakdowns within each domain reveal distinct research priorities and applications. Based on 738 original studies. Created with Canva (https://www.canva.com).

To clarify the scope and application of these models, studies were classified by design and stated objectives into either neurodevelopment in health (65%, 351/543) or neurodevelopment in disease (35%, 192/543), as shown in Figure 4.

We define “neurodevelopment in health” as studies that use non‐pathological cellular material to investigate typical developmental processes. While not explicitly disease‐focused, such models may serve as platforms for subsequent modeling of neuropathology. In contrast, “neurodevelopment in disease” refers to studies explicitly designed to model pathological processes.

#### 2.1.1 Neurodevelopment in Health

Physiological neurodevelopment is governed by a coordinated progression of molecular and cellular events that establish the structural and functional architecture of the brain [77, 78]. These include early patterning cues [84], the generation of diverse neural lineages [85] and the formation of region‐specific circuits [86] that underpin cognition and behavior [87]. Understanding these normative processes is critical for defining developmental benchmarks and contextualizing disease‐associated deviations.

**FIGURE 5:**
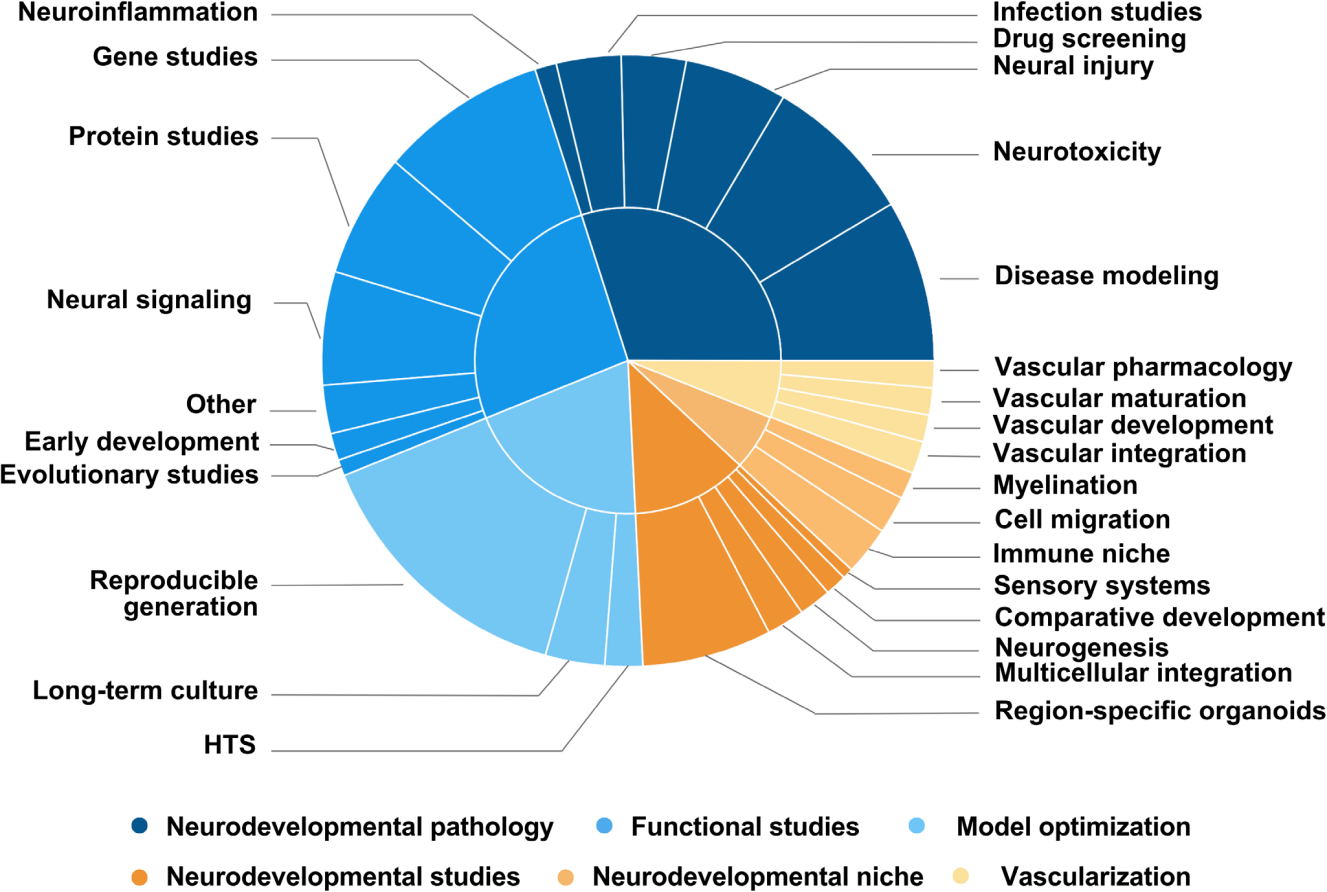
Organoids as models of neurodevelopment in health. Sunburst plot illustrating the thematic breakdown of organoid‐based neurodevelopment studies. Neurodevelopmental pathology modeling was the most represented theme (29.9%), followed by functional studies (26.2%), model optimization (19.7%), basic neurodevelopmental studies (18.2%), and vascularization (6.0%). Based on 351 original studies. HTS, high‐throughput screening. Created with Canva (https://www.canva.com).

In this context, organoids have been widely applied to investigate the molecular and cellular mechanisms underlying early brain development [11, 27]. A total of 351 original studies were identified that utilized cerebral organoids to model neurodevelopment in health (Figure 5). Neurodevelopmental pathology modeling, focused on the transition from physiological to pathological states, was the most represented category (29.9%, 105/351) [48, 58, 62, 77, 81, 88, 89, 90, 91, 92, 93, 94, 95, 96, 97, 98, 99, 100, 101, 102, 103, 104, 105, 106, 107, 108, 109, 110, 111, 112, 113, 114, 115, 116, 117, 118, 119, 120, 121, 122, 123, 124, 125, 126, 127, 128, 129, 130, 131, 132, 133, 134, 135, 136, 137, 138, 139, 140, 141, 142, 143, 144, 145, 146, 147, 148, 149, 150, 151, 152, 153, 154, 155, 156, 157, 158, 159, 160, 161, 162, 163, 164, 165, 166, 167, 168, 169, 170, 171, 172, 173, 174, 175, 176, 177, 178, 179, 180, 181, 182, 183, 184, 185, 186, 187], followed by functional studies (26.2%, 92/351) [41, 63, 188, 189, 190, 191, 192, 193, 194, 195, 196, 197, 198, 199, 200, 201, 202, 203, 204, 205, 206, 207, 208, 209, 210, 211, 212, 213, 214, 215, 216, 217, 218, 219, 220, 221, 222, 223, 224, 225, 226, 227, 228, 229, 230, 231, 232, 233, 234, 235, 236, 237, 238, 239, 240, 241, 242, 243, 244, 245, 246, 247, 248, 249, 250, 251, 252, 253, 254, 255, 256, 257, 258, 259, 260, 261, 262, 263, 264, 265, 266, 267, 268, 269, 270, 271, 272, 273, 274, 275, 276, 277], model optimization (19.7%, 69/351) [25, 40, 59, 278, 279, 280, 281, 282, 283, 284, 285, 286, 287, 288, 289, 290, 291, 292, 293, 294, 295, 296, 297, 298, 299, 300, 301, 302, 303, 304, 305, 306, 307, 308, 309, 310, 311, 312, 313, 314, 315, 316, 317, 318, 319, 320, 321, 322, 323, 324, 325, 326, 327, 328, 329, 330, 331, 332, 333, 334, 335, 336, 337, 338, 339, 340, 341, 342, 343], neurodevelopmental studies (12.2%, 43/351) [24, 43, 47, 60, 83, 344, 345, 346, 347, 348, 349, 350, 351, 352, 353, 354, 355, 356, 357, 358, 359, 360, 361, 362, 363, 364, 365, 366, 367, 368, 369, 370, 371, 372, 373, 374, 375, 376, 377, 378, 379, 380, 381], neurodevelopmental niche (6.0%, 21/351) [80, 382, 383, 384, 385, 386, 387, 388, 389, 390, 391, 392, 393, 394, 395, 396, 397, 398, 399, 400, 401], and vascularization (6.0%, 21/351) [402, 403, 404, 405, 406, 407, 408, 409, 410, 411, 412, 413, 414, 415, 416, 417, 418, 419, 420, 421, 422].

##### 2.1.1.1 Neurodevelopmental Pathology

Organoid‐based studies investigating the transition from typical development to early‐stage pathology accounted for nearly one‐third (29.9%, 105/351) [48, 58, 62, 77, 81, 88, 89, 90, 91, 92, 93, 94, 95, 96, 97, 98, 99, 100, 101, 102, 103, 104, 105, 106, 107, 108, 109, 110, 111, 112, 113, 114, 115, 116, 117, 118, 119, 120, 121, 122, 123, 124, 125, 126, 127, 128, 129, 130, 131, 132, 133, 134, 135, 136, 137, 138, 139, 140, 141, 142, 143, 144, 145, 146, 147, 148, 149, 150, 151, 152, 153, 154, 155, 156, 157, 158, 159, 160, 161, 162, 163, 164, 165, 166, 167, 168, 169, 170, 171, 172, 173, 174, 175, 176, 177, 178, 179, 180, 181, 182, 183, 184, 185, 186, 187]. These models were employed to explore mechanisms that predispose the developing brain to disease, including genetic susceptibility, environmental toxicants, pharmacological exposures, early neural injury and infection. Six primary application areas were identified (Figure 5): disease modeling (28.6%, 30/105) [48, 58, 62, 88, 89, 90, 91, 92, 93, 94, 95, 96, 97, 98, 99, 100, 101, 102, 103, 104, 105, 106, 107, 108, 109, 110, 111, 112, 113, 114], neurotoxicity (26.7%, 28/105) [77, 81, 115, 116, 117, 118, 119, 120, 121, 122, 123, 124, 125, 126, 127, 128, 129, 130, 131, 132, 133, 134, 135, 136, 137, 138, 139, 140], neural injury (18.1%, 19/105) [141, 142, 143, 144, 145, 146, 147, 148, 149, 150, 151, 152, 153, 154, 155, 156, 157, 158, 159], drug screening (11.4%, 12/105) [160, 161, 162, 163, 164, 165, 166, 167, 168, 169, 170, 171], infection studies (11.4%, 12/105) [172, 173, 174, 175, 176, 177, 178, 179, 180, 181, 182, 183], and neuroinflammation (3.8%, 4/105) [184, 185, 186, 187]. (Study distributions are shown in Figure 5, with detailed information available via the supplementary *OrganoidMap* web application and Excel file *OrganoidMap.xlsx*.)

Disease modeling represented the largest application area (28.6%, 30/105) [48, 58, 62, 88, 89, 90, 91, 92, 93, 94, 95, 96, 97, 98, 99, 100, 101, 102, 103, 104, 105, 106, 107, 108, 109, 110, 111, 112, 113, 114], with cerebral, midbrain, hypothalamic, thalamic and forebrain organoids derived from hESCs and hiPSCs used to recapitulate neurodevelopment and early‐onset pathologies. Genetic perturbations, either via patient‐derived iPSCs or CRISPR‐based editing [102], targeted key disease‐associated genes, including *SHANK3* [100], *CDK6* [98], *ARID1B* [58], and *PCDH12* [105]. These studies revealed region‐specific cytoarchitecture [100], transcriptional fidelity [100], synaptic activity [111], and captured both cell‐autonomous and non‐cell‐autonomous mechanisms across multiple developmental trajectories [107]. Organoids were employed to interrogate processes such as cortical folding [98], interneuron migration [105], mitochondrial dysfunction [91], and excitatory/inhibitory imbalance. Multimodal readouts, including single‐cell RNA sequencing (scRNA‐seq), proteomics, live imaging, and electrophysiology, were commonly used to deepen mechanistic insight.

Organoid‐based neurotoxicity studies accounted for 26.7% (28/105) [77, 81, 115, 116, 117, 118, 119, 120, 121, 122, 123, 124, 125, 126, 127, 128, 129, 130, 131, 132, 133, 134, 135, 136, 137, 138, 139] of neurodevelopmental pathology research, evaluating the developmental impact of ethanol [115], nicotine [81], anesthetics (e.g., sevoflurane) [125], methamphetamine [118], cadmium [121, 130], nanomaterials, endocrine disruptors, and other neurotoxicants. Cerebral organoids were predominantly used, with additional studies incorporating region‐specific platforms [140] or multi‐lineage co‐cultures. Readouts included impaired neurogenesis [140], apoptosis [121], oxidative stress [132], disrupted synaptogenesis [130], and transcriptomic dysregulation, often linked to mechanistic pathways such as PI3K‐AKT‐mTORC1 [116], Notch [122], Wnt [139], and NLRP3 [187]. Functional endpoints were supported by calcium imaging [129], multi‐electrode array (MEA) recordings, lipidomics, and, in select cases, integration with organ‐on‐chip platforms to simulate chronic exposure or improve nutrient diffusion.

Studies investigating neural injury (18.1%, 19/105) [141, 142, 143, 144, 145, 146, 147, 148, 149, 150, 151, 152, 153, 154, 155, 156, 157, 158, 159] focused predominantly on regenerative potential and circuit‐level integration following trauma [147, 153, 159]. Cerebral, cortical, and pituitary organoids were employed in both *ex vivo* and *in vivo* settings to model injury and transplantation outcomes [159]. Several studies demonstrated engraftment of organoid‐derived tissue into mouse, rat, or primate brains, with evidence of axonal extension, synaptic formation, and functional integration into host circuitry [144, 152, 154]. Targeted applications included restoration of corticospinal pathways, visual cortex reinnervation, and repair of spinal cord lesions. Some studies further explored pharmacological preconditioning, trophic factor delivery, and modulation of neuroinflammation to enhance graft survival and function [157].

Drug screening studies (11.4%, 12/105) [160, 161, 162, 163, 164, 165, 166, 167, 168, 169, 170, 171] applied organoids to evaluate therapeutic compounds, gene delivery vectors, and seizure liability in the context of neurodevelopment. Cerebral, cortical [169], thalamic [164], neurovascular [161], and choroid plexus [165, 170] organoids were employed to test agents targeting genetic disorders, excitability syndromes, or infectious neuropathology. Notable applications included high‐throughput adeno‐associated virus (AAV) capsid screening [163], analysis of phenylbutyrate‐induced oligodendrogenesis [166], cannabinoid signaling and assessment of anti‐seizure efficacy through MEA‐based functional profiling [167]. Some platforms incorporated dynamic perfusion, cerebrospinal fluid secretion assays, or personalized iPSC lines to evaluate drug permeability, mechanisms of action, and variability in patient response.

Models of central nervous system (CNS) infection (11.4%, 12/105) [172, 173, 174, 175, 176, 177, 178, 179, 180, 181, 182, 183] used cerebral, forebrain, and choroid plexus organoids to study viral and parasitic exposures during early development. These included investigations into SARS‐CoV‐2 [174, 177, 180, 181], HSV1 [183], Enterovirus D68 [175], and *Trypanosoma brucei* [179], with a focus on viral tropism, cell‐type susceptibility, and induction of innate immune pathways. Several studies incorporated microglia [177], endothelial cells, or pericytes to simulate blood‐brain or blood‐cerebrospinal fluid (CSF) barrier dysfunction and to evaluate host–pathogen interactions. Antiviral strategies were also tested, including CRISPR‐based editing of HSV‐1 and pharmacologic inhibition of SARS‐CoV‐2 replication, demonstrating the feasibility of therapeutic interrogation in a 3D human context.

Across neurodevelopmental pathology studies, pathological phenotypes were often modeled in isolation, with limited resolution of cell–cell and circuit‐level interactions. In addition, reliance on a small number of cell lines and limited *in vivo* validation further constrain translational relevance.

##### 2.1.1.2 Functional Studies

Organoid‐based functional studies (26.2%, 92/351) [41, 63, 188, 189, 190, 191, 192, 193, 194, 195, 196, 197, 198, 199, 200, 201, 202, 203, 204, 205, 206, 207, 208, 209, 210, 211, 212, 213, 214, 215, 216, 217, 218, 219, 220, 221, 222, 223, 224, 225, 226, 227, 228, 229, 230, 231, 232, 233, 234, 235, 236, 237, 238, 239, 240, 241, 242, 243, 244, 245, 246, 247, 248, 249, 250, 251, 252, 253, 254, 255, 256, 257, 258, 259, 260, 261, 262, 263, 264, 265, 266, 267, 268, 269, 270, 271, 272, 273, 274, 275, 276, 277] were broadly categorized into gene function (33.7%, 31/92) [41, 188, 189, 190, 191, 192, 193, 194, 195, 196, 197, 198, 199, 200, 201, 202, 203, 204, 205, 206, 207, 208, 209, 210, 211, 212, 213, 214, 215, 216, 217], protein function (25.0%, 23/92) [63, 218, 219, 220, 221, 222, 223, 224, 225, 226, 227, 228, 229, 230, 231, 232, 233, 234, 235, 236, 237, 238, 239], neural signaling (22.8%, 21/92) [240, 241, 242, 243, 244, 245, 246, 247, 248, 249, 250, 251, 252, 253, 254, 255, 256, 257, 258, 259, 260], early brain development (5.4%, 5/92) [261, 262, 263, 264, 265], evolutionary studies (3.3%, 3/92) [266, 267, 268], and other applications (9.8%, 9/92) [269, 270, 271, 272, 273, 274, 275, 276, 277] (See Figure 5; detailed study information is available via the *OrganoidMap* web application and the supplementary Excel file: *OrganoidMap.xlsx*).

These studies leveraged human‐derived cerebral, cortical [191, 210, 215], forebrain [189, 214] and region‐specific organoids [209], primarily from hESCs and hiPSCs, to dissect cell‐intrinsic and network‐level processes underpinning brain development [214], homeostasis [193] and vulnerability to disease.

Gene‐focused studies employed organoids to interrogate transcriptional [208, 209] and epigenetic [204] regulation during corticogenesis. These models were used to explore the function of key transcription factors (e.g., SOX1 [194], GLI3 [208], ZNF558 [199]), chromatin regulators (e.g., MPP8 [205] and MORC2A [205]), and noncoding RNA species such as lncRNAs, circRNAs, and miRNAs. Functional readouts included transcriptomic profiling, clonal lineage tracing [216], enhancer mapping [214], and perturbation assays [192] across developmental timepoints. Several studies applied high‐resolution techniques—such as scRNA‐seq, nanopore‐based isoform profiling, and lentiMPRA [214] to reconstruct gene regulatory networks [214], chromatin dynamics [215], and splicing programs [207]. Comparative approaches incorporating non‐human primate organoids [203] enabled insights into species‐specific gene functions and evolutionarily divergent regulatory elements.

Protein‐function studies (25.0%, 23/92) [63, 218, 219, 220, 221, 222, 223, 224, 225, 226, 227, 228, 229, 230, 231, 232, 233, 234, 235, 236, 237, 238, 239] investigated intracellular signaling pathways central to neurodevelopment, including mTOR [237], GSK3β [227], FGF [218], DRD1 [232] and insulin receptor pathways [223]. These models illuminated mechanisms governing progenitor proliferation [222], neuronal migration [219], apoptosis [227], and synaptic development [239]. Experimental approaches included CRISPR‐mediated knockouts [236], phospho‐proteomics and bioelectronic modulation platforms, including ion pumps and neuromodulation arrays. Notably, several studies employed proteomics‐integrated readouts to characterize AMPylation [221], phosphorylation, and receptor‐mediated signaling cascades in human organoids.

Neural signaling studies (22.8%, 21/92) [240, 241, 242, 243, 244, 245, 246, 247, 248, 249, 250, 251, 252, 253, 254, 255, 256, 257, 258, 259, 260] focused on the emergence and regulation of electrophysiological activity [252], neurotransmission [245] and circuit maturation [249]. Organoids were used to model serotonergic [241] and GABAergic [254, 260] pathways, as well as neuromodulatory influences from hormones [247] (e.g. androgens, glucocorticoids) and psychoactive compounds [241, 256] (e.g., 5‐MeO‐DMT, flavonoids). Functional platforms included high‐density microelectrode arrays (HD‐CMOS MEA) [259], cantilever‐based electrodes [259], patch‐clamp recordings, and integrated optical and electrophysiological readouts [252]. Studies reported spontaneous oscillatory activity, phase‐locked firing, and coordinated bursts across cortical domains, as well as axon tract formation and circuit‐level plasticity.

Early brain development studies (5.4%, 5/92) [261, 262, 263, 264, 265] leveraged cerebral and neuronal [263] organoids to examine human‐specific progenitor dynamics [265], early neuronal network formation [263], and thyroid hormone signaling [265]. These models recapitulated features of first‐trimester cortical neurogenesis, including layer‐specific neuron formation and hormone responsiveness.

Evolutionary studies (3.3%, 3/92) [266, 267, 268] used organoids derived from human and non‐human [268] primate iPSCs to investigate the molecular basis of neocortical expansion, basal progenitor proliferation, and the emergence of gyrification. Key findings centered on regulatory transitions involving the Robo‐Notch‐Dll1 axis [267] and primate‐specific genes such as ARHGAP11B [266] and TBC1D3 [268].

Other applications (9.8%, 9/92) [269, 270, 271, 272, 273, 274, 275, 276, 277] spanned areas including mitochondrial function [270], intercellular communication via extracellular vesicles [277], lipid metabolism [272], organoid heterogeneity profiling, and protein trafficking [273]. These studies employed cutting‐edge modalities such as spatial metabolomics [272], single‐organoid mass spectrometry [274], and live imaging of intracellular dynamics [273], underscoring the expanding functional repertoire of organoid‐based systems.

In these functional studies, specific limitations included reliance on single iPSC lines, editing‐induced mosaicism and limited validation of predicted regulatory interactions. Functional outcomes were frequently inferred from transcriptomic or static phenotypic data, with relatively few studies incorporating dynamic or long‐term functional assessments.

##### 2.1.1.3 Model Optimization

Model optimization studies comprised 19.7% (69/351) [25, 40, 59, 278, 279, 280, 281, 282, 283, 284, 285, 286, 287, 288, 289, 290, 291, 292, 293, 294, 295, 296, 297, 298, 299, 300, 301, 302, 303, 304, 305, 306, 307, 308, 309, 310, 311, 312, 313, 314, 315, 316, 317, 318, 319, 320, 321, 322, 323, 324, 325, 326, 327, 328, 329, 330, 331, 332, 333, 334, 335, 336, 337, 338, 339, 340, 341, 342, 343] of the included literature and were primarily focused on enhancing reproducibility (73.9%, 51/69) [25, 40, 59, 278, 279, 280, 281, 282, 283, 284, 285, 286, 287, 288, 289, 290, 291, 292, 293, 294, 295, 296, 297, 298, 299, 300, 301, 302, 303, 304, 305, 306, 307, 308, 309, 310, 311, 312, 313, 314, 315, 316, 317, 318, 319, 320, 321, 322, 323, 324, 325], supporting long‐term culture (15.9%, 11/69) [326, 327, 328, 329, 330, 331, 332, 333, 334, 335, 336] and enabling high‐throughput screening (HTS, 10.1%, 7/69) [337, 338, 339, 340, 341, 342, 343] (Figure 5; *OrganoidMap* web application and *OrganoidMap.xlsx*).

Reproducibility‐focused efforts emphasized scalable [294, 384], consistent protocols for cerebral [318], cortical [283], forebrain [286], and region‐specific organoids [285], with advances in microfluidic platforms [292, 301], Matrigel‐free systems [291], acoustic bioreactors, and geometrically defined scaffolds. These strategies improved organoid uniformity, reduced necrotic core formation, and facilitated batch‐to‐batch consistency. Long‐term culture studies extended viability beyond 200 days [327], enabling investigation of neuronal maturation [331], synaptic integration [335], and aging‐related processes [334]. Innovations included dynamic perfusion [333], scaffold‐supported systems, and integrated real‐time imaging platforms. HTS models supported automated generation and multiplexed readouts [340], incorporating midbrain [337] and cortical [338] organoids for disease modeling and drug evaluation. Platforms employed pooled donor libraries, assembloid formation, and light‐sheet imaging to enhance scalability.

Limitations specific to model optimization included persistent variability in organoid architecture despite protocol improvements as well as incomplete recapitulation of late‐stage maturation and interregional connectivity.

##### 2.1.1.4 Neurodevelopmental Studies

Neurodevelopmental studies (12.3%, 43/351) [24, 43, 47, 60, 83, 344, 345, 346, 347, 348, 349, 350, 351, 352, 353, 354, 355, 356, 357, 358, 359, 360, 361, 362, 363, 364, 365, 366, 367, 368, 369, 370, 371, 372, 373, 374, 375, 376, 377, 378, 379, 380, 381] were categorized into region‐specific organoid models (55.8%, 24/43) [24, 60, 83, 345, 346, 347, 348, 349, 350, 351, 352, 353, 354, 355, 356, 357, 358, 359, 360, 361, 362, 363, 364, 365, 366, 367, 368, 369, 370, 371, 372, 373, 374, 375, 376, 377, 378, 379], multicellular integration (16.3%, 7/43) [47, 348, 353, 354, 356, 365, 380], neurogenesis (14.0%, 6/43) [43, 359, 375, 376, 378, 381], comparative neurodevelopment (9.3%, 4/43) [352, 361, 366, 374] and sensory systems (4.7%, 2/43) [344, 360] (Figure 5; *OrganoidMap* web application and *OrganoidMap.xlsx*).

Region‐specific organoids modeled, among others, the midbrain [345, 357, 363], thalamus [350], hypothalamus [355], pituitary [362], striatum [364], hippocampus [370], cerebellum [379], and spinal cord [377] to reconstruct spatially patterned differentiation, lineage trajectories, and circuit emergence. These models supported investigations into cortical folding [346], neurotransmitter dynamics, and compartmentalized development, also incorporating genetic perturbation or disease‐associated mutations [368]. Multicellular integration studies leveraged assembloids [47, 353, 354] and co‐cultures [348] to model circuit‐level connectivity across regions and germ layers, enabling interrogation of axon pathfinding, striatal projections, and cortico‐retinal interactions. Neurogenesis studies explored human‐specific progenitor behaviors [43], clonal lineage dynamics [376], and radial glia heterogeneity using live imaging, single‐cell lineage tracing, and polarity‐switching organoid systems. Comparative studies integrated primate [361, 374], rodent [352], and hybrid [361] iPSC models to investigate species‐specific regulatory features in corticogenesis and astrocyte maturation [366]. Sensory system models focused on early visual system patterning and bilateral optic vesicle development within self‐organizing organoids [344, 360].

Limitations across neurodevelopmental studies included restricted modeling of late‐stage maturation as well as variability in lineage fidelity and regional identity.

##### 2.1.1.5 Neurodevelopmental Niche

Neurodevelopmental niche studies (6.0%, 21/351) [80, 382, 383, 384, 385, 386, 387, 388, 389, 390, 391, 392, 393, 394, 395, 396, 397, 398, 399, 400, 401] focused on the immune niche (42.9%, 9/21) [80, 382, 383, 384, 385, 386, 387, 388, 389], cell migration (33.3%, 7/21) [390, 391, 392, 393, 394, 395, 396], and myelination (23.8%, 5/21) [397, 398, 399, 400, 401] (Figure 5; *OrganoidMap* web application and *OrganoidMap.xlsx*).

Immune niche models incorporated iPSC‐derived [382, 383, 389] or primary [382, 388] microglia into cerebral [80, 387] or retinal [389] organoids, enabling investigations of neuroinflammation [385], synaptic pruning [389], and cytokine signaling [385] in disease‐relevant contexts (e.g., maternal immune activation). Studies employed co‐cultures [383], assembloids, and multi‐omic tools [388] to capture microglial maturation and gene regulation within brain‐like environments. Migration‐focused platforms used assembloids [391, 394], forebrain organoids [394] as well as live imaging [390] to explore human‐specific interneuron positioning, extracellular matrix (ECM) modulation [395], and cortical integration [393]. Myelination models implemented long‐term cultures [398] and dorsal‐ventral assembloids [399] to induce oligodendrogenesis and myelin formation, with relevance to demyelinating disorders and therapeutic screening [397].

Key limitations included inconsistent glial maturation and batch‐dependent variability in immune and oligodendrocyte incorporation across models.

##### 2.1.1.6 Vascularization

Vascularization studies (6.0%, 21/351) [402, 403, 404, 405, 406, 407, 408, 409, 410, 411, 412, 413, 414, 415, 416, 417, 418, 419, 420, 421, 422] were grouped into vascular integration (28.6%, 6/21) [403, 407– 409, 411, 413], vascular development (23.8%, 5/21) [404, 405, 406, 410, 415], vascular maturation (23.8%, 5/21) [402, 414, 416, 417, 422], and vascular pharmacology (23.8%, 5/21) [412, 418, 419, 420, 421] (Figure 5; *OrganoidMap* web application and *OrganoidMap.xlsx*). Integration‐focused models incorporated endothelial and pericyte co‐cultures, assembloids, and microfluidic systems to reconstruct neurovascular units and blood–brain barrier (BBB)‐like interfaces [403, 407, 408, 409, 411, 413]. These platforms enabled exploration of neuronal‐endothelial cross‐talk [406], microglial signaling and circuit formation, with several models achieving spatially organized, transcriptionally aligned vascularization. Developmental studies leveraged directed differentiation and VEGF‐based protocols to model vasculogenesis and angiogenesis within organoids, reproducing perineural plexus organization and vascular signaling gradients [404, 405, 406, 410, 415]. Maturation approaches included *in vivo* engraftment [402, 416], soft microfluidic scaffolds [405], and long‐term co‐culture [402] to support vessel functionality, synaptic integration, and multi‐lineage survival [402, 414, 416, 417, 422]. Pharmacological platforms evaluated BBB permeability, receptor‐mediated transport, and neurovascular toxicity using patient‐derived assembloids, high‐throughput imaging, and CRISPR perturbation [412, 418, 419, 420, 421].

Specific limitations included reliance on non‐CNS endothelial sources, lack of perfusion, adaptive vascular remodeling, and reduced control over vascular architecture as well as long‐term integration.

##### 2.1.1.7 Comparative Advantages, Limitations, and Future Directions

Cerebral organoids offer an ethically accessible and experimentally tractable platform to model human brain development in unprecedented detail. Unlike 2D *in vitro* cultures, organoids enable self‐organization, multi‐lineage interactions and spatial patterning reflective of early neurodevelopment [45, 64, 126, 423, 424]. Compared to *in vivo* rodent models, they better recapitulate human‐specific features—including progenitor diversity, cortical expansion, and transcriptional programs—while circumventing interspecies differences and invasive experimentation [425]. For the first time, these models permit direct, human‐focused interrogation of developmental events previously inaccessible in non‐primate systems [11, 50]. However, despite their widespread adoption in studies of neurodevelopment in health, cerebral organoids remain constrained by a set of fundamental and recurring limitations [4, 5, 27, 33, 51]. Most prominently, batch‐to‐batch variability [274, 302, 310] and limited reproducibility [33, 162, 316] persist even under ostensibly standardized protocols, complicating the establishment of robust developmental benchmarks and cross‐study comparability. Variability in lineage specification and spatial patterning remains substantial, resulting in inconsistent representation of progenitor zones, cortical layers, and non‐neuronal cell populations.

Region‐specific maturation is frequently incomplete, with developmental trajectories often stalling at early fetal‐like stages. Moreover, the absence of standardized developmental staging systems limits temporal alignment with in vivo neurodevelopment, complicating efforts to benchmark maturation trajectories or compare results across platforms. In addition, axis specification (e.g., anterior–posterior, dorsal–ventral) remains poorly controlled in many protocols, particularly those employing unguided or minimally patterned systems. Importantly, these challenges intensify as model complexity increases. Efforts to improve regional patterning introduce additional sensitivity to morphogen timing and concentration. Functional studies are constrained by limited electrophysiological maturity, short culture windows and heterogeneous circuit formation, with relatively few platforms demonstrating stable, reproducible functional outputs over time. Long‐term cultures, while enabling advanced readouts, may also exhibit transcriptional and epigenetic drift, raising concerns about fidelity to late‐stage developmental programs. Expansion of cellular composition, including astrocytes, oligodendrocytes, microglia, and endothelial cells, adds further sources of variability, particularly in co‐culture and assembloid systems, where integration efficiency and maturation state remain difficult to control. Vascularized organoids, while promising, continue to face limitations related to perfusion, endothelial identity and long‐term stability. Furthermore, reliance on a narrow set of pluripotent stem cell lines, often lacking diversity in sex, ancestry, or genotype, may limit generalizability and obscure biologically meaningful variation. Together, these findings indicate that even baseline neurodevelopmental organoid models are not neutral reference systems, but instead represent biologically informative yet methodologically constrained platforms. To fully realize their potential for defining normative human neurodevelopment, organoid systems will require coordinated advances in protocol harmonization, quantitative benchmarking, and controlled integration of functional and cellular niches.

#### 2.1.2 Neurodevelopment in Disease

Disruptions to early brain development, whether genetic, metabolic, infectious, or environmental, can lead to neurodevelopmental disorders (NDDs) [426, 427], a diverse group of early‐onset conditions that affect 15%–20% of children worldwide [72]. These include autism spectrum disorder (ASD), intellectual disability, epilepsy, and attention‐deficit/hyperactivity disorder (ADHD), among others [428, 429, 430, 431, 432, 433, 434, 435].

**FIGURE 6:**
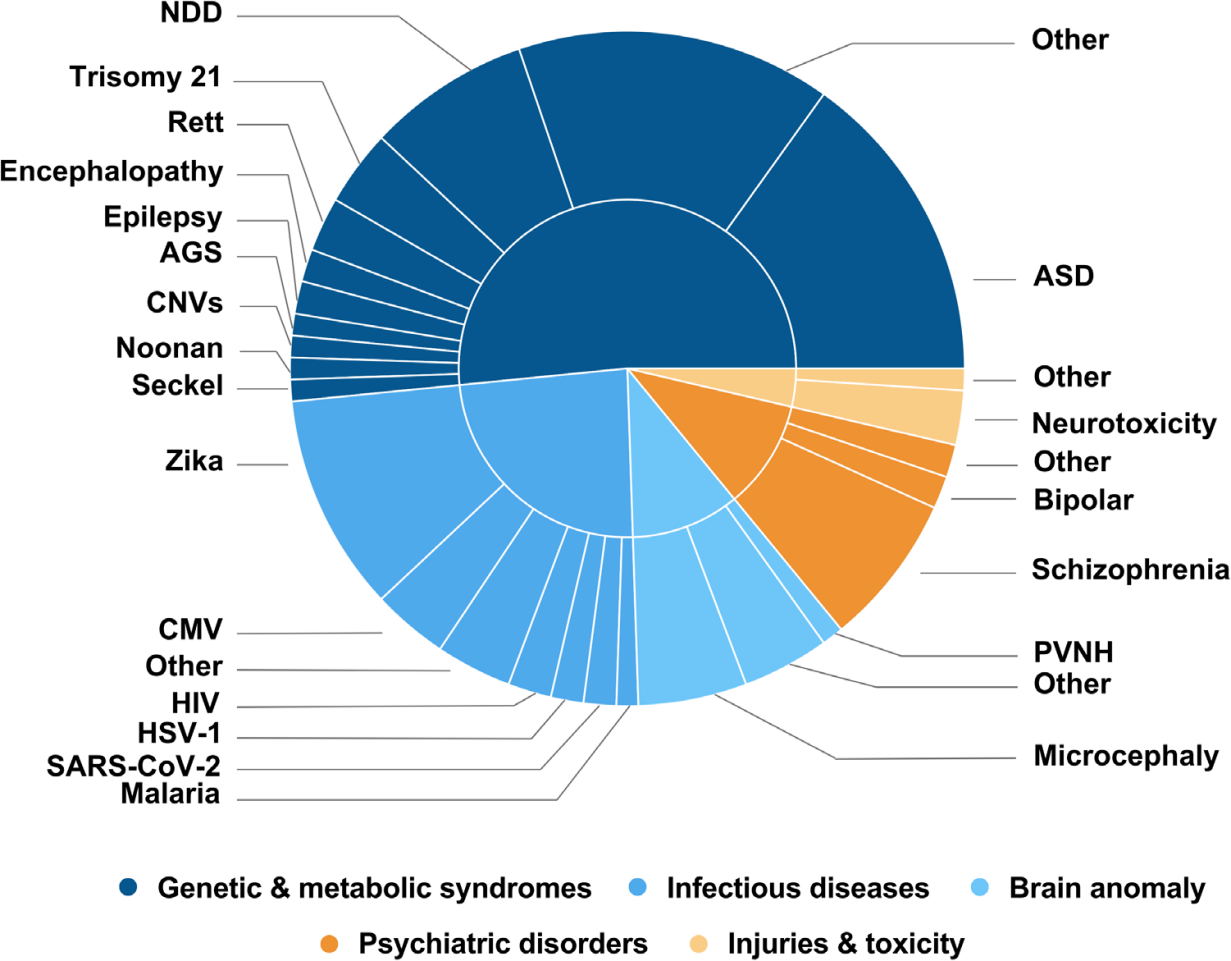
Organoids as models of neurodevelopment in disease. Sunburst plot illustrating organoid use across major disease categories. Genetic and metabolic disorders account for the majority (51.6%), followed by infectious (23.9%), psychiatric (10.4%), structural (10.4%), and injury/toxicity‐related (3.6%) conditions. Based on 192 original studies. ASD, autism spectrum disorder; Bipolar, bipolar disorder; CMV, cytomegalovirus; CNVs, copy number variants; HIV, human immunodeficiency virus; HSV‐1, herpes simplex virus type 1; NDD, neurodevelopmental disorder; Noonan, Noonan syndrome; PVNH, periventricular nodular heterotopia; Rett, Rett syndrome; SARS‐CoV‐2, severe acute respiratory syndrome coronavirus 2; Seckel, Seckel syndrome; Zika, Zika virus. Created with Canva (https://www.canva.com).

Cerebral organoids offer a human‐specific platform to model NDDs, enabling mechanistic studies of early brain development and disease‐relevant phenotypes [436]. Among 192 studies identified, 52.1% (100/192) focused on genetic and metabolic disorders [437, 438, 439, 440, 441, 442, 443, 444, 445, 446, 447, 448, 449, 450, 451, 452, 453, 454, 455, 456, 457, 458, 459, 460, 461, 462, 463, 464, 465, 466, 467, 468, 469, 470, 471, 472, 473, 474, 475, 476, 477, 478, 479, 480, 481, 482, 483, 484, 485, 486, 487, 488, 489, 490, 491, 492, 493, 494, 495, 496, 497, 498, 499, 500, 501, 502, 503, 504, 505, 506, 507, 508, 509, 510, 511, 512, 513, 514, 515, 516, 517, 518, 519, 520, 521, 522, 523, 524, 525, 526, 527, 528, 529, 530, 531, 532, 533, 534, 535, 536], 24.0% (46/192) on infection‐related conditions [537, 538, 539, 540, 541, 542, 543, 544, 545, 546, 547, 548, 549, 550, 551, 552, 553, 554, 555, 556, 557, 558, 559, 560, 561, 562, 563, 564, 565, 566, 567, 568, 569, 570, 571, 572, 573, 574, 575, 576, 577, 578, 579, 580, 581, 582], 10.4% (20/192) on psychiatric disorders [583, 584, 585, 586, 587, 588, 589, 590, 591, 592, 593, 594, 595, 596, 597, 598, 599, 600, 601, 602], 9.9% (19/192) on structural malformations [603, 604, 605, 606, 607, 608, 609, 610, 611, 612, 613, 614, 615, 616, 617, 618, 619, 620, 621], and 3.6% (7/192) on acquired injuries or toxic exposures (Figure 6) [622, 623, 624, 625, 626, 627, 628].

##### 2.1.2.1 Genetic and Metabolic Disorders

Genetic and metabolic NDDs were the most frequently modeled subgroup, accounting for 52.0% (100/192) of studies [437, 438, 439, 440, 441, 442, 443, 444, 445, 446, 447, 448, 449, 450, 451, 452, 453, 454, 455, 456, 457, 458, 459, 460, 461, 462, 463, 464, 465, 466, 467, 468, 469, 470, 471, 472, 473, 474, 475, 476, 477, 478, 479, 480, 481, 482, 483, 484, 485, 486, 487, 488, 489, 490, 491, 492, 493, 494, 495, 496, 497, 498, 499, 500, 501, 502, 503, 504, 505, 506, 507, 508, 509, 510, 511, 512, 513, 514, 515, 516, 517, 518, 519, 520, 521, 522, 523, 524, 525, 526, 527, 528, 529, 530, 531, 532, 533, 534, 535, 536]. These conditions are commonly linked to single‐gene mutations, chromosomal abnormalities, or metabolic disruptions that impair early brain development. ASD was the most extensively studied diagnosis (29.0%, 29/100) [437, 438, 439, 440, 441, 442, 443, 444, 445, 446, 447, 448, 449, 450, 451, 452, 453, 454, 455, 456, 457, 458, 459, 460, 461, 462, 463, 464, 465], followed by general NDDs (15.0%, 15/100) [466, 467, 468, 469, 470, 471, 472, 473, 474, 475, 476, 477, 478, 479, 480], trisomy 21 (7.0%, 7/100) [481, 482, 483, 484, 485, 486, 487, 488], Rett syndrome (5.0%, 5/100) [489, 490, 491, 492, 493], and other genetic and metabolic conditions [494, 495, 496, 497, 498, 499, 500, 501, 502, 503, 504, 505, 506, 507, 508, 509, 510, 511, 512, 513, 514, 515, 516, 517, 518, 519, 520, 521, 522, 523, 524, 525, 526, 527, 528, 529, 530, 531, 532, 533, 534, 535, 536] (Figure 6; *OrganoidMap* web application and *OrganoidMap.xlsx*), all commonly associated with disrupted neurogenesis, cortical patterning and synaptic organization. Most studies employed hiPSC‐derived cerebral organoids or assembloids to examine gene‐specific effects across neuronal, glial and progenitor populations. Frequently investigated genes included CHD8, PTEN, DYRK1A and SHANK3, as well as structural variants such as 16p11.2 deletions and duplications [440, 442, 450, 451, 458, 461, 482]. These models revealed consistent alterations in progenitor proliferation, interneuron migration, and excitatory/inhibitory (E/I) balance. Environmental exposures, such as valproic acid, were also incorporated to examine gene–environment interactions [452]. Multi‐omic approaches and advanced imaging enabled detailed phenotyping of morphogenesis and molecular dysregulation.

Microglia were integrated in four studies including Aicardi‐Goutières syndrome, revealing altered cholesterol metabolism and phagocytic activity [476, 477, 478, 535]. However, the broader absence of immune system components limited investigation of inflammation‐mediated phenotypes.

Metabolic disorders, including epilepsy, encephalopathy, Sandhoff disease, and Cockayne syndrome, were modeled in 8.0% (8/100) of studies and frequently involved disruptions in anabolic pathways relevant to the neurovascular unit [498, 499, 506, 507, 513, 523, 524, 527].

Despite growing interest in genetically defined NDDs, modeling efforts remained focused on ASD. In contrast, other high‐burden or genetically tractable conditions—such as intellectual disability, Tourette syndrome, Dravet syndrome, trisomy 21, and Rett syndrome—were less represented. This imbalance highlights the need for broader inclusion of both rare and complex disorders to fully capture mechanistic diversity.

##### 2.1.2.2 Infectious Disease‐Associated Disorders

Congenital infections are a major environmental contributor to NDDs. Infection‐related models accounted for 23.9% (46/192) of studies [537, 538, 539, 540, 541, 542, 543, 544, 545, 546, 547, 548, 549, 550, 551, 552, 553, 554, 555, 556, 557, 558, 559, 560, 561, 562, 563, 564, 565, 566, 567, 568, 569, 570, 571, 572, 573, 574, 575, 576, 577, 578, 579, 580, 581, 582], with Zika virus (ZIKV) most frequently studied (43.5%) [538, 539, 543, 546, 549, 552, 554, 555, 567, 568, 569, 570, 573, 574, 576, 578, 579, 580, 581, 582], followed by human cytomegalovirus (HCMV, 15.2%) [545, 547, 550, 551, 559, 565, 566], HIV (8.6%) [541, 557, 558, 575], HSV‐1 (6.5%), and SARS‐CoV‐2 (6.5%) [553, 572, 577]. Other pathogens included measles virus, malaria, and Usutu virus [540, 542, 561, 564] (Figure 6; *OrganoidMap* web application and *OrganoidMap.xlsx*).

ZIKV models used hiPSC‐derived organoids or neural progenitor cultures to examine viral tropism, vertical transmission, and regional vulnerability. These platforms supported antiviral screening (e.g., betulinic acid), high‐resolution imaging of viral pathology, and one investigation into ZIKV's oncolytic potential. Two studies incorporated microglia, revealing divergent immune responses to virus infections [542, 552].

HCMV models demonstrated impaired cortical development and altered neuronal differentiation [545, 547, 550, 551, 559, 565, 566]. Mechanistic studies explored nitric oxide as a modulator of viral toxicity and profiled viral entry pathways across developing neural cell types [566]. Overall, infectious disease models revealed that congenital infections can impair neurodevelopment through both direct cytopathic effects and secondary disruptions in immune signaling, transcriptional regulation, and metabolism.

Despite this, modeling efforts remained heavily focused on ZIKV, with limited exploration of other pathogens. The minimal use of immune‐competent systems restricted insight into host‐pathogen interactions.

##### 2.1.2.3 Psychiatric Disorders

Psychiatric disorders, including schizophrenia (SCZ), bipolar disorder (BD), and ADHD, affect roughly one in eight individuals worldwide. These conditions arise from multifactorial genetic, epigenetic, and environmental interactions, many of which shape early brain development.

Of the studies reviewed, 10.4% (20/192) focused on psychiatric conditions [583, 584, 585, 586, 587, 588, 589, 590, 591, 592, 593, 594, 595, 596, 597, 598, 599, 600, 601, 602], with SCZ comprising 70.0% (14/20) [583, 584, 585, 586, 587, 588, 589, 590, 591, 592, 593, 594, 595, 596], followed by BD (15.0%) [597, 598, 599] and other psychiatric disorders (15.0%) [600, 601, 602] (Figure 6; *OrganoidMap* web application and *OrganoidMap.xlsx*). Most studies used hiPSC‐derived organoids to model early neurodevelopmental trajectories and assess the impact of risk‐associated variants.

Variants in MAD1L1 and NRXN1 were linked to disruptions in progenitor dynamics and cortical organization [593, 594]. Multi‐omic approaches, including scRNA‐seq, TWAS, and co‐expression analyses, prioritized risk loci and candidate mechanisms. Perturbations were reported in TNF signaling, striatal development, and lineage‐specific transcriptional networks [588].

However, limited maturity and underdeveloped circuitry constrain the utility of these models for studying adolescent‐onset disorders. Study distribution remained heavily weighted toward SCZ, with minimal attention to BD, ADHD, or broader psychiatric phenotypes.

##### 2.1.2.4 Structural Brain Malformations

Structural malformations result from early disruptions in neurodevelopmental processes such as neuronal proliferation, migration, and cortical patterning. These conditions, including microcephaly, periventricular nodular heterotopia (PVNH), and lissencephaly, were modeled in 9.8% (19/192) of studies [603, 604, 605, 606, 607, 608, 609, 610, 611, 612, 613, 614, 615, 616, 617, 618, 619, 620, 621] (Figure 6; *OrganoidMap* web application and *OrganoidMap.xlsx*).

Microcephaly was the most studied (52.6%, 10/19), often linked to mutations in WDR62, ASPM, and KNL1, which regulate progenitor maintenance and cortical expansion [603, 607, 608, 610, 614, 616, 617, 619, 620, 621]. Models of Nijmegen breakage syndrome and AUTS2 variants implicated deficits in DNA damage response and transcriptional control [608, 614]. Additionally, one study used time‐lapse imaging and single‐cell transcriptomics to investigate progenitor subtype dynamics [613].

PVNH was modeled in 10.5% (2/19) of studies using DCHS1 or FAT4‐mutant iPSC lines, successfully recapitulating migrational defects [605, 615]. While these models yielded valuable insights into morphogenetic disruptions, incomplete regional specification, and limited maturation constrained the interpretation of later‐stage phenotypes. The relative scarcity of studies in this area underscores the need for broader modeling of complex brain malformations.

##### 2.1.2.5 Acquired Injuries and Toxic Exposures

A small proportion of studies (3.6%, 7/192) [622, 623, 624, 625, 626, 627, 628] investigated acquired injuries and neurotoxic exposures as contributors to NDDs (Figure 6; *OrganoidMap* web application and *OrganoidMap.xlsx*). These included environmental toxicants, pharmacologic agents, and metabolic disturbances that impair early brain development.

Ethanol exposure—modeling fetal alcohol spectrum disorders—was shown to disrupt neurogenesis and was validated in fetal neurons [622]. Additional studies examined bisphenol A (BPA), tetrodotoxin (TTX), and hydroxynorketamine (HNK), revealing transcriptomic dysregulation and impaired synaptic function. These models provided insights into early developmental vulnerability but were limited by short‐term viability, exposure variability, and reduced tissue complexity. The small number of studies highlights a broader underrepresentation of environmentally driven NDD models.

##### 2.1.2.6 Comparative Advantages, Limitations, and Future Directions

Cerebral organoids provide a uniquely human‐relevant platform for modeling NDDs, particularly those arising from early genetic, metabolic, or environmental perturbations. Their ability to capture patient‐specific genotypes, early developmental trajectories, and cell‐typeresolved phenotypes has enabled mechanistic interrogation of disorders that are difficult to model in animal systems, including autism spectrum disorder, congenital infections, and monogenic developmental syndromes. Despite these advantages, the application of organoid systems to NDD research faces several domain‐specific limitations. Most disease phenotypes are modeled against an incompletely defined developmental baseline, complicating interpretation of whether observed alterations reflect pathological deviation or normal variability in organoid maturation. This challenge is particularly relevant for disorders characterized by subtle shifts in progenitor dynamics, neuronal subtype balance, or circuit formation. In addition, the predominance of early fetal‐like states limits the ability to model later‐emerging features of NDDs, including synaptic refinement, activity‐dependent plasticity, and circuit stabilization. Current disease modeling efforts also show a pronounced imbalance in disorder representation. ASD and Zika virus infection account for a substantial proportion of studies, whereas many high‐burden, genetically tractable, or environmentally driven NDDs remain underrepresented. This limits comparative insight across disease classes and constrains generalization of methodological advances. Moreover, most studies rely on a limited number of donor‐derived iPSC lines, often without systematic inclusion of polygenic risk, sex‐specific effects, or population diversity, restricting the capacity to capture the heterogeneity that characterizes many NDDs. Functional interpretation remains another key bottleneck. While transcriptomic and morphological phenotyping are widely employed, fewer studies incorporate longitudinal or network‐level functional readouts. As a result, disease‐relevant alterations in excitatory–inhibitory balance, synaptic integration, or circuit dynamics are often inferred indirectly rather than measured directly. Similarly, limited incorporation of microglia, vasculature, and immune signaling constrains modeling of inflammation‐associated phenotypes that are increasingly recognized as contributors to NDD pathogenesis. Moving forward, progress in NDD modeling will require organoid platforms that better support defined developmental benchmarking, increased donor and disorder diversity and more consistent functional validation. Modular systems, including assembloids and bioengineered platforms, offer opportunities to capture inter‐regional and immuneneural interactions that are central to many neurodevelopmental disorders. Together, these advances will be essential for translating organoid‐based insights into robust mechanistic understanding and, ultimately, clinically relevant intervention strategies.

### 2.2 Cerebral Organoids in Neurodegeneration

Neurodegenerative diseases (NDs) are a leading cause of disability and death worldwide, affecting approximately 15% of the global population [629, 630]. Their burden is particularly pronounced in aging societies, where prevalence continues to rise due to increasing life expectancy [631]. Alzheimer's disease (AD) and Parkinson's disease (PD) are the most common neurodegenerative disorders, although many less common pathologies remain understudied and mechanistically unresolved [632]. As the societal and economic costs of neurodegeneration escalate [632], there is an urgent need for human‐relevant models that can recapitulate disease‐specific mechanisms and guide therapeutic discovery. Here, cerebral organoids offer a 3D, physiologically relevant system for modeling brain disorders [23]. Over the past decade, they have been increasingly applied to study the molecular and cellular underpinnings of neurodegenerative disease.

**FIGURE 7:**
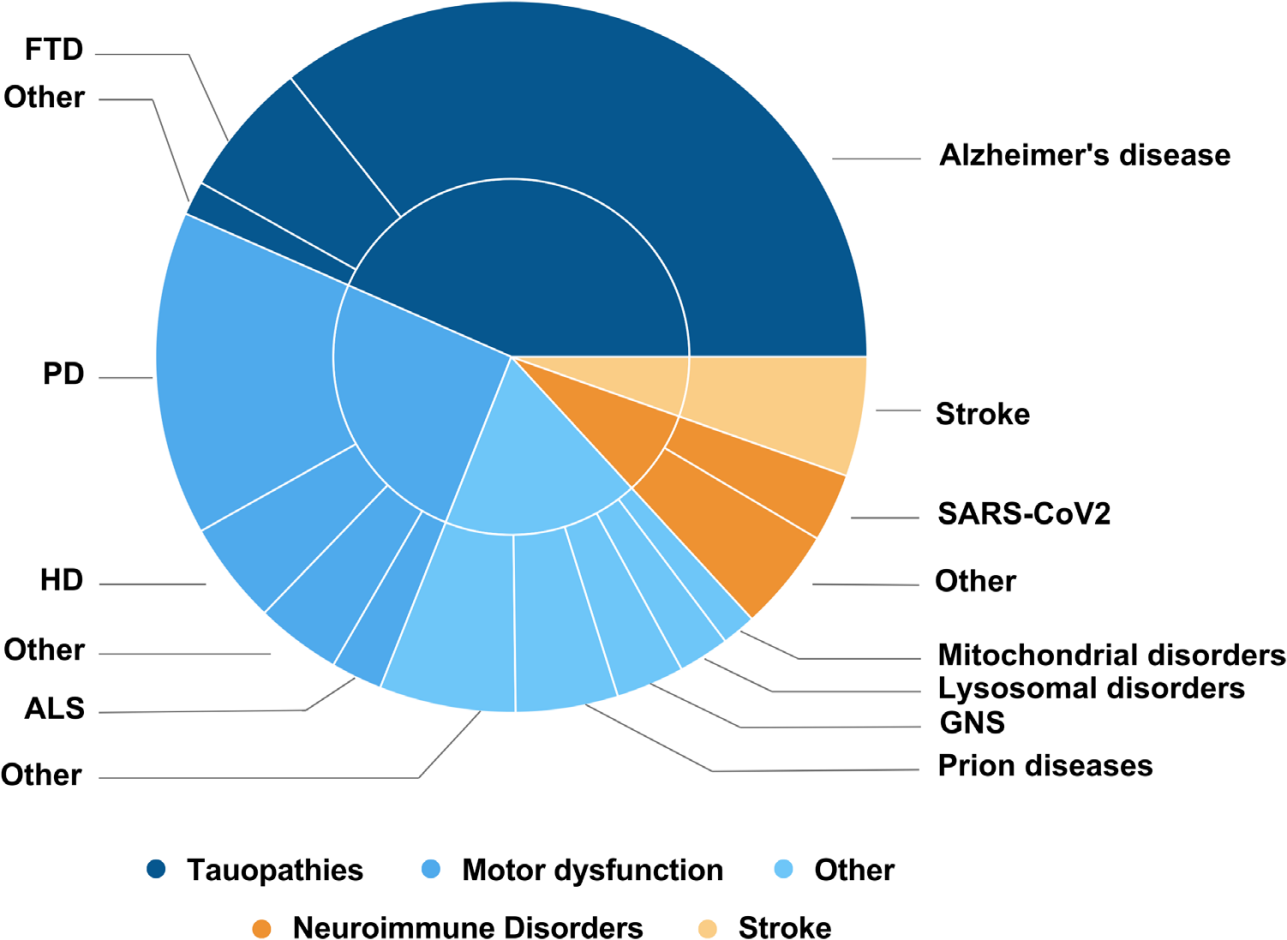
Organoids as models of neurodegeneration. Sunburst plot illustrating organoid use across neurodegenerative pathologies. Tauopathies were most represented (43.4%), followed by motor dysfunction disorders (25.6%), neuroimmune disorders (7.8%), stroke (5.5%), and other conditions, including genetic, metabolic, and prion diseases (17.8%). Based on 129 original studies. ALS, amyotrophic lateral sclerosis; FTD, frontotemporal dementia; HD, Huntington's disease; GNS, genetic neurological syndromes; PD, Parkinson's disease; SARS‐CoV‐2, severe acute respiratory syndrome coronavirus 2. Created with Canva (https://www.canva.com).

A total of 129 original studies were identified that utilized cerebral organoids to model NDs (Figure 7) [633, 634, 635, 636, 637, 638, 639, 640, 641, 642, 643, 644, 645, 646, 647, 648, 649, 650, 651, 652, 653, 654, 655, 656, 657, 658, 659, 660, 661, 662, 663, 664, 665, 666, 667, 668, 669, 670, 671, 672, 673, 674, 675, 676, 677, 678, 679, 680, 681, 682, 683, 684, 685, 686, 687, 688, 689, 690, 691, 692, 693, 694, 695, 696, 697, 698, 699, 700, 701, 702, 703, 704, 705, 706, 707, 708, 709, 710, 711, 712, 713, 714, 715, 716, 717, 718, 719, 720, 721, 722, 723, 724, 725, 726, 727, 728, 729, 730, 731, 732, 733, 734, 735, 736, 737, 738, 739, 740, 741, 742, 743, 744, 745, 746, 747, 748, 749, 750, 751, 752, 753, 754, 755, 756, 757, 758, 759, 760, 761]. Tauopathies were the most frequently represented category (43.4%, 56/129) [633, 634, 635, 636, 637, 638, 639, 640, 641, 642, 643, 644, 645, 646, 647, 648, 649, 650, 651, 652, 653, 654, 655, 656, 657, 658, 659, 660, 661, 662, 663, 664, 665, 666, 667, 668, 669, 670, 671, 672, 673, 674, 675, 676, 677, 678, 679, 680, 681, 682, 683, 684, 685, 686, 687, 688], followed by motor dysfunction disorders (25.6%, 33/129) [689, 690, 691, 692, 693, 694, 695, 696, 697, 698, 699, 700, 701, 702, 703, 704, 705, 706, 707, 708, 709, 710, 711, 712, 713, 714, 715, 716, 717, 718, 719, 720, 721], genetic, metabolic, and prion diseases (12.4%, 16/129) [722, 723, 724, 725, 726, 727, 728, 729, 730, 731, 732, 733, 734, 735, 736, 737], neuroimmune disorders (7.8%, 10/129) [738, 739, 740, 741, 742, 743, 744, 745, 746, 747], stroke (5.5%, 7/129) [748, 749, 750, 751, 752, 753, 754], and other conditions (5.5%, 7/129) [755, 756, 757, 758, 759, 760, 761].

#### 2.2.1 Tauopathies

Tauopathies were the most extensively modeled group, reflecting the high prevalence and clinical burden of AD and related disorders [762]. These diseases are characterized by pathological tau aggregation into neurofibrillary tangles, leading to neuronal dysfunction and cell death [763]. Among the 56 tauopathy‐focused studies, AD accounted for 82.1% (46/56), followed by frontotemporal dementia (FTD, 14.3%, 8/56) [679, 680, 681, 682, 683, 684, 685, 686], and studies exploring general tau aggregation mechanisms (3.6%, 2/56) [687, 688] (Figure 7; *OrganoidMap* web application and *OrganoidMap.xlsx*).

Most studies utilized hiPSC‐derived cerebral organoids to recapitulate hallmark features such as amyloid‐β plaques and tau tangles. These systems enabled modeling at physiological gene expression levels, providing patient‐specific platforms for both mechanistic exploration and therapeutic screening. Fourteen studies leveraged organoids for drug testing [633, 634, 635, 636, 637, 638, 639, 640, 641, 642, 643, 644, 645, 646, 682], underscoring their translational potential.

Genetic manipulation approaches included both CRISPR‐Cas9 and viral transfection systems, allowing precise modeling of mutation‐driven pathology [633, 647, 648, 649, 650, 651, 652, 653, 654, 655, 680, 681, 684, 687]. Notable findings include the identification of p25/Cdk5 signaling in tauopathy progression [680] and ApoE4‐associated dysfunction in AD‐derived cells [647]. Down syndrome‐derived organoids were used to study the role of trisomy 21 in early‐onset AD [657].

The incorporation of microglia enhanced modeling of neuroinflammatory cascades and their interaction with tau pathology [647, 677]. Chimeric organoids combining healthy and AD‐derived cells facilitated investigation of cell‐type‐specific contributions in a shared environment [656]. However, the absence of fully functional vasculature and immune complexity continues to limit the *in vivo* relevance of these systems [656].

#### 2.2.2 Motor Dysfunction Disorders

Motor dysfunction disorders—such as PD, Huntington's disease (HD), and amyotrophic lateral sclerosis (ALS)—are characterized by the progressive loss of motor neurons or dopaminergic circuits [764, 765]. PD was the most studied condition in this group (57.6%, 19/33) [683, 689, 690, 691, 692, 693, 694, 695, 696, 697, 698, 699, 700, 701, 702, 703, 704, 705, 706], consistent with its status as the second most prevalent ND, affecting over 10 million individuals worldwide [766]. HD and ALS represented 18.2% (6/33) [707, 708, 709, 710, 711, 712] and 12.1% (4/33) [713, 714, 715, 716], respectively, with the remaining studies focusing on broader Parkinsonian predispositions [717, 718, 719] (Figure 7; *OrganoidMap* web application and *OrganoidMap.xlsx*).

hiPSC‐derived midbrain organoids were central to PD research, enabling modeling of dopaminergic neuron loss and Lewy body formation [683, 689, 690, 691, 692, 693, 694, 695, 696, 697, 698, 699, 700, 701]. Several studies used these models for high‐throughput neuroprotective drug screening [691, 693, 697, 701, 702, 705, 706, 721]. To improve reproducibility, one study employed midbrain floor plate neural progenitor cells (mfNPCs) instead of direct differentiation from hiPSCs [692]. A notable approach used chimeric organoids incorporating neurons from both healthy and PD donors to minimize batch effects and highlight cell‐intrinsic phenotypes [698]. One study employed rat postnatal olfactory bulb tissue to reduce animal use, although species‐specific differences limited its translational relevance [697].

In HD models, telencephalic organoids were developed using mosaic cultures of HD and control hiPSCs to study cell‐type‐specific pathology [709]. Striatonigral assembloids enabled modeling of projection neuron dysfunction and the cortico‐striatal circuit, though these required long‐term culturing to recapitulate advanced neurodegeneration [711]. ALS models similarly benefited from the addition of microglia, allowing exploration of inflammation‐driven disease progression [716].

Several studies investigating genetic predisposition used cerebral organoids to analyze excitotoxicity, neurophysiological properties and stress responses [717, 718, 719, 767]. While these models exhibited *in vivo*‐like characteristics, they continued to face challenges including limited maturity, biological complexity, and batch variability [717, 718, 719, 767].

#### 2.2.3 Neuroimmune Disorders and Stroke

Neuroimmune disorders, defined by immune‐mediated neuronal injury [768], accounted for 7.8% (10/129) of studies. SARS‐CoV‐2‐associated neurodegeneration was the most commonly modeled (40%) [738, 739, 740, 741], followed by multiple sclerosis (MS, 30%) [742, 743, 744], with HSV‐1 [745], *Mycoplasma fermentans* [746], and immune‐driven aging comprising the remaining 10%[747] (Figure 7; *OrganoidMap* web application and *OrganoidMap.xlsx*). Organoid platforms were employed to examine virus‐host interactions, neuroinflammation and antiviral drug responses [738, 739, 740, 741, 745]. MS models specifically investigated oligodendrocyte differentiation and lesion formation using hiPSC‐derived cerebral organoids [742– 744].

A key study combined forebrain organoids with microglia to explore macroglia‐microglia interactions [744]. Despite this advance, limitations in cellular maturation and inter‐sample variability remain barriers to broader adoption.

Stroke was modeled in 5.4% (7/129) of studies, with most using hiPSC‐derived cerebral organoids [748, 749, 750, 751, 752]. Considering that stroke remains one of the leading causes of death worldwide, it is markedly underrepresented in cerebral organoid research. One study employed rat cortical spheroids to reduce animal use, although species differences limited human relevance [753]. A critical barrier across all stroke models is the lack of a functional BBB, vascular perfusion and immune components, factors essential for replicating ischemic pathophysiology and systemic responses [748, 749, 750, 751, 752, 753, 754].

#### 2.2.4 Genetic, Metabolic, and Prion Disease Modeling

A wide range of rare neurodegenerative diseases were modeled using cerebral organoids, including progressive myoclonic epilepsy [722, 723], GM1 gangliosidosis [724], Niemann‐Pick disease [725], ataxia‐telangiectasia [726, 727], Batten disease [728], and Creutzfeldt–Jakob disease [729, 730, 731, 732, 733, 737] (Figure 7; *OrganoidMap* web application and *OrganoidMap.xlsx*). These studies explored mechanisms, such as synaptic dysfunction [722, 723, 734], mitochondrial impairment [726, 727], protein misfolding [729, 730, 731, 732, 733, 737, 758], and gene therapy delivery [724]. While these models recapitulated key aspects of disease pathology, they were limited by donor variability, reduced biological complexity, and developmental immaturity.

Beyond classical NDs, organoid systems were used to study related neurodegenerative contexts, including traumatic brain injury [755], spaceflight‐induced brain changes [756], and environmental neurotoxicity [757]. These applications demonstrate the versatility of cerebral organoids for modeling diverse neurological insults.

#### 2.2.5 Comparative Advantages, Limitations, and Future Directions

Cerebral organoids offer a human‐relevant platform for modeling neurodegenerative diseases, enabling mechanistic studies in patient‐derived genetic backgrounds with physiological expression levels. Their 3D architecture and cellular diversity allow interrogation of early pathogenic processes, such as protein misfolding, oxidative stress responses, and glial activation, under conditions that better reflect human neurobiology than traditional animal models [769]. However, their application to neurodegeneration remains limited by key domain‐specific constraints. Most platforms reflect early fetal stages, lacking age‐associated features such as proteostatic decline, telomere shortening, or cumulative oxidative damage. Consequently, while organoids often reproduce select molecular hallmarks, e.g., amyloid‐β aggregation in AD or dopaminergic vulnerability in PD, they rarely capture the full spectrum of progressive, late‐onset pathology. Tau pathology, for instance, is typically modeled in isolation from downstream neurofibrillary spread or circuit‐level degeneration. Functional integration of supporting systems remains incomplete. Microglia were incorporated in only 3.9% of studies [647, 677, 716, 744, 760], despite their central role in neuroinflammation and disease progression, substantially limiting the modeling of immune‐mediated neurodegenerative mechanisms. Vascularized platforms remain rare and often lack perfusion [705], restricting studies of BBB dysfunction, drug delivery, or vascular contributions to pathology. These limitations are particularly acute in diseases where neuroimmune and vascular interactions are central to progression, such as MS, stroke and AD. Disease coverage across organoid‐based ND research is disproportionate. Tauopathies and PD dominate, while ALS, Huntington's disease, prion disorders, and cerebrovascular insults remain underrepresented. Additionally, many studies rely on a narrow set of iPSC lines without systematic inclusion of sex, ancestry, or polygenic risk‐limiting generalizability. Electrophysiological and network‐level phenotyping remains variable across platforms, further complicating interpretation of synaptic or circuit‐level dysfunction. Advancing organoid‐based neurodegeneration research will require the development of long‐lived, vascularized and immune‐competent models that support aging trajectories and later‐stage phenotypes. Engineered systems, such as assembloids, organoid‐on‐chip platforms, and inducible aging models, offer promising routes to address these gaps. Together with protocol standardization and high‐throughput, multimodal phenotyping, these innovations will be critical to improving reproducibility, capturing disease heterogeneity, and enabling translational applications in neurodegenerative disease research.

### 2.3 Cerebral Organoids in Neuro‐Oncology

Neuro‐oncological disorders are among the most lethal and clinically intractable conditions, with brain tumors exhibiting high morbidity, poor prognosis, and limited treatment options [770]. GBM, the most aggressive primary brain tumor, has a median survival of less than 15 months despite standard therapy [771], while brain metastases affect up to 20% of patients with advanced cancers such as breast, lung, and melanoma [772, 773, 774]. As the global cancer burden continues to rise, driven by aging populations and extended survival, there is growing demand for human‐specific models that can recapitulate tumor biology and therapeutic response in the brain. Cerebral organoids provide a physiologically relevant, 3D platform to study tumor initiation, invasion, and TME interactions in a human neural context [775].

**FIGURE 8:**
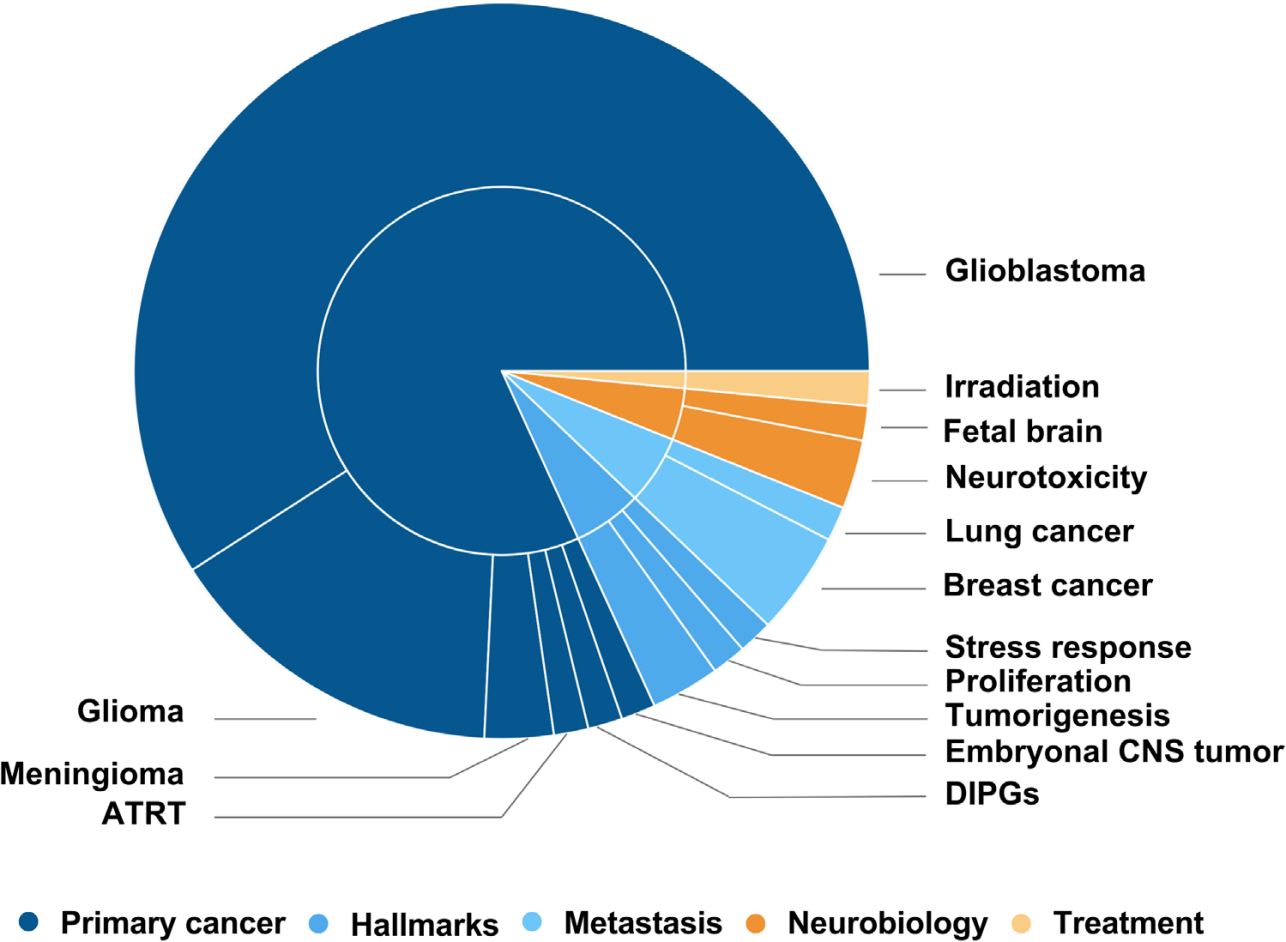
Organoids as models of neuro‐oncology. Sunburst plot illustrating organoid use in neuro‐oncology research. Most studies focused on primary CNS tumors (81.8%), followed by brain metastases (6.0%), cancer hallmarks (6.0%), neurobiological mechanisms (4.6%), and treatment strategies (1.5%). Based on 66 original studies. ATRT, atypical teratoid/rhabdoid tumor; CNS, central nervous system; DIPG, diffuse intrinsic pontine glioma. Created with Canva (https://www.canva.com).

Over the past decade, organoid‐based systems have been increasingly applied to neuro‐oncology research (Figure 8). A total of 66 original studies were identified that utilized cerebral organoids to model brain cancer. Most studies focused on primary CNS tumors (81.8%, 54/66) [70, 71, 776, 777, 778, 779, 780, 781, 782, 783, 784, 785, 786, 787, 788, 789, 790, 791, 792, 793, 794, 795, 796, 797, 798, 799, 800, 801, 802, 803, 804, 805, 806, 807, 808, 809, 810, 811, 812, 813, 814, 815, 816, 817, 818, 819, 820, 821, 822, 823, 824, 825, 826, 827], with fewer addressing brain metastases (6.0%, 4/66) [73, 74, 75, 828], cancer hallmarks (6.0%, 4/66) [829, 830, 831, 832], neurobiological mechanisms (4.6%, 3/66) [833, 834, 835], or therapeutic strategies (1.5%, 1/66) [836].

#### 2.3.1 Primary CNS Cancer

Primary CNS tumors represented the dominant focus within neuro‐oncology organoid studies [70, 71, 776, 777, 778, 779, 780, 781, 782, 783, 784, 785, 786, 787, 788, 789, 790, 791, 792, 793, 794, 795, 796, 797, 798, 799, 800, 801, 802, 803, 804, 805, 806, 807, 808, 809, 810, 811, 812, 813, 814, 815, 816, 817, 818, 819, 820, 821, 822, 823, 824, 825, 826, 827], with gliomas modeled in 90.7% (49/54) of cases and GBM alone comprising 72.2% (39/54) (Figure 8; *OrganoidMap* web application and *OrganoidMap.xlsx*). This emphasis reflects the clinical urgency surrounding GBM [837], an aggressive malignancy with limited treatment options and poor survival [838]. Organoid models included patient‐derived GBM organoids [70, 789, 791, 796], genetically engineered cerebral organoids [779, 780, 784], co‐cultures [70, 777, 787, 807, 812], and assembloids such as GLICO [71, 785, 798, 805, 818], each offering distinct insights into tumor initiation [781, 810, 831], invasion [70, 74, 786, 814, 816], lineage plasticity [803], and therapeutic resistance [782, 785, 791, 798, 801, 802, 816]. Studies explored key disease features including outer radial glia (oRG) reactivation [798], transcriptional GBM states [787, 798], and tumor–neuron interactions [798]. CRISPR‐based editing [780, 781], hydrogel microfluidics, and multi‐omic profiling were employed to model genetic drivers (e.g., TP53 [800], PTEN [794, 800], EGFRvIII [813]), examine glioma stem cell behavior [71], and test therapeutic efficacy [71]. Co‐culture platforms enabled high‐throughput compound screening [71, 794] and real‐time imaging in a human neural context, while assembloid systems offered enhanced spatial fidelity for tumorhost integration [818]. Despite this progress, modeling efforts were largely restricted to high‐grade gliomas. Lower‐grade gliomas, atypical teratoid/rhabdoid tumors (ATRT) [776], diffuse intrinsic pontine glioma (DIPG) [777], and embryonal CNS tumors [778] were each represented in only a single study. Meningiomas, among the most prevalent adult brain tumors [839], appeared in just 3.7% (2/54) of studies, highlighting a persistent gap in organoid‐based models for non‐glial CNS cancers.

Limitations specific to primary CNS cancer models included reduced representation of non‐GBM tumor types [776] and limited use of primary tissue [71, 806, 816, 824]. Across platforms, a trade‐off persisted between preserving tumor heterogeneity and achieving scalability [796, 798, 801], with challenges in standardization and cross‐comparability further constraining translational relevance.

#### 2.3.2 Brain Metastases

Despite representing the most common form of adult brain tumors [840, 841, 842], secondary brain cancers were modeled in only 6.0% (4/66) of studies [73, 74, 75, 828] (Figure 8; *OrganoidMap* web application and *OrganoidMap.xlsx*). Three studies focused on breast‐to‐brain metastases [73, 74, 75] using co‐cultures or organoid‐on‐a‐chip platforms to investigate tumor adaptation, small extracellular vesicle (sEV) dynamics and tissue‐specific interactions. One study examined broader metastatic mechanisms in cerebral organoids derived from hESCs [828]. These models demonstrated *in vivo*‐like architecture and translational potential for drug screening. However, these models remain limited by low biological complexity, particularly in immune cell integration and tumor vascularization, as well as specimen‐dependent heterogeneity and short culture lifespan, highlighting the need for more representative and durable platforms for metastasis research.

#### 2.3.3 Cancer Hallmarks

Four studies (6.0%, 4/66) modeled cancer hallmarks such as tumorigenesis (2/4) [831, 832], proliferation (1/4) [829] and stress response (1/4) [830] (Figure 8; *OrganoidMap* web application and *OrganoidMap.xlsx*). Platforms included co‐cultures, hESC‐derived cerebral organoids [831] and GLICO assembloids [832], with applications ranging from BCAT1‐driven chromosomal instability and stress granule modulation to CRISPR‐induced tumorigenesis and DHFR‐targeted BTIC self‐renewal. While these models enabled mechanistic insights and patient‐specific profiling, they were limited by the absence of a tumor‐immune microenvironment and high material requirements.

#### 2.3.4 Cancer Neurobiology

Three studies (4.6%, 3/66) [833, 834, 835] investigated neurobiological mechanisms, including BBB permeability [833], neurotoxicity [835], and fetal brain organization [834] (Figure 8; *OrganoidMap* web application and *OrganoidMap.xlsx*). Models employed hESC‐ and hiPSC‐derived organoids [834] or primary human tissue [833, 835] to assess vincristine‐induced damage, off‐target drug toxicity, and region‐specific developmental programs. While platforms preserved key neuroanatomical features and supported CRISPR‐based perturbation, limitations included lack of mature BBB properties, time‐intensive protocols, and dependence on fetal tissue availability.

#### 2.3.5 Cancer Treatment

Only one study (1.5%, 1/66) [836] directly evaluated therapeutic strategy (Figure 8; *OrganoidMap* web application and *OrganoidMap.xlsx*), using co‐cultures of cerebral organoids and patient‐derived glioma stem‐like cells to assess FLASH radiotherapy. However, findings remain preliminary, with limited systemic context and a lack of clinical cross‐validation restricting translational applicability.

#### 2.3.6 Comparative Advantages, Limitations, and Future Directions

Cerebral organoids provide a human‐specific [781], ethically viable platform to model brain tumor biology, enabling spatially resolved analysis of tumor–host interactions, lineage dynamics, and therapy resistance. Compared to 2D cultures [843, 844], they retain cytoarchitecture and tumor heterogeneity; relative to rodent models [785, 790, 792, 794, 819], they offer species‐relevant insight without ethical or translational constraints. Despite these advantages, several limitations remain. Most models lack vasculature, immune components and systemic context [789, 790], limiting their capacity to capture angiogenesis, neuroinflammation and pharmacokinetics. Functional modeling of the BBB remains underdeveloped, constraining studies on drug penetration and vascular niche interactions. Adaptive immune components are absent, precluding evaluation of immunotherapies, immune evasion, or tumor immunoediting. Tumor evolution and clonal dynamics are rarely captured, with most models representing static snapshots rather than dynamic disease trajectories. Current studies are heavily biased toward glioblastoma. Lower‐grade gliomas, non‐glial tumors, such as meningioma and pediatric brain cancers remain underrepresented. Similarly, brain metastases, despite being the most common adult brain tumors, have received minimal attention. The lack of extracellular matrix control limits mechanobiological modeling, while the limited use of primary patient tissue restricts representation of inter‐ and intra‐tumoral heterogeneity. Another trade‐off persists between model complexity and scalability [815], with challenges in standardization [796, 807], cross‐comparability, and long‐term culture. Critically, systematic validation against clinical outcomes is lacking, and few studies assess whether organoid‐derived responses predict patient trajectories. Overall, this field remains in its infancy, with fewer than 70 studies published between 2014 and 2024, most within the past 5 years. Nevertheless, cerebral organoids hold immense potential, particularly for modeling brain metastases, a growing global health burden with limited preclinical tools. Expanded tumor representation, incorporation of vasculature, immune niches, and longitudinal benchmarking will be essential to realize the translational promise of these systems. Further, integration of organoids into co‐clinical trial pipelines and validation against therapeutic response data will be the next critical steps in moving these models toward functional precision oncology.

## 3 Discussion

Over the past decade, cerebral organoids have emerged as a transformative model system in neuroscience, enabling the study of species‐specific brain development in health and disease. This systematic review presents the first cross‐domain synthesis of cerebral organoid applications spanning neurodevelopment, neurodegeneration, and neuro‐oncology, encompassing 738 original studies. The current landscape shows a strong emphasis on human‐derived systems, with over 95% of studies employing hiPSCs, hESCs, or primary human tissue. Their growing adoption reflects both scientific potential and ethical responsibility, providing a scalable, human‐relevant alternative to animal models and traditional 2D systems, while supporting the principles of the 3Rs (Replacement, Reduction and Refinement). Across domains, organoids have enabled unprecedented access to early human brain development, cellular lineage trajectories, and disease pathophysiology in a 3D context. Neurodevelopmental studies dominate the field, advancing our understanding of cortical patterning, progenitor dynamics, and genetic neurodevelopmental disorders. While still comparatively underrepresented, neurodegeneration and neuro‐oncology applications are expanding, with studies increasingly leveraging patient‐derived iPSCs, CRISPR perturbations, and multi‐omic profiling. These models offer platforms for drug testing, mechanistic discovery, and translational research positioning organoids as a critical tool in modern neuroscience.

To support this synthesis, we developed *OrganoidMap*—an open‐source, interactive web application that centralizes curated data and enables researchers to explore, filter, and visualize the cerebral organoid literature across domains. To our knowledge, it is the first open‐access platform to provide structured, searchable access to the field at this scale, encompassing 738 original studies. Unlike traditional reviews that rely on static figures or summary tables, *OrganoidMap* allows real‐time querying by disease context, model type, cell source, and methodological features. The platform addresses major challenges in the field: consolidating fragmented literature, offering standardized terminology through searchable keywords, and enabling direct model comparison. By making structured data broadly accessible, *OrganoidMap* advances transparency and sets a new benchmark for reproducible, open science in the organoid research community.

Example use cases include identifying underrepresented research areas (e.g., brain metastases or pediatric gliomas), assessing protocol variation (e.g., use of co‐cultures or gene editing tools), and selecting appropriate models for translational or mechanistic studies. Researchers can benchmark existing approaches, uncover technical trends, and make more informed experimental decisions.

Crucially, *OrganoidMap* is designed as a living resource. While the current version reflects data extracted up to 09 October 2024, future updates will integrate community feedback and expand the metadata to include functional readouts and reproducibility indicators.

By democratizing access to the cerebral organoid literature, *OrganoidMap* serves not only as a reference but also as a practical tool for accelerating discovery, improving standardization, and supporting translational impact across the global neuroscience community.

### 3.1 Bridging Neuroscience and Biomaterials Innovation

While cerebral organoids are not traditional biomaterials, they increasingly function as bioengineerable platforms, offering living, self‐organizing systems with emergent structural and functional properties. Beyond their significance for neuroscience, this review and *OrganoidMap* hold broader relevance for the materials science and bioengineering communities. Organoids exhibit layered organization, cellular heterogeneity, and dynamic remodeling, positioning them as tunable systems with conceptual parallels to synthetic scaffolds, biohybrid constructs, and engineered living materials. As organoids are integrated into biofabrication workflows, organoid‐on‐chip technologies, and functionalized extracellular matrices, the demand for standardized, interoperable, and transparent model information becomes increasingly urgent. Future directions will require the development of benchmarking frameworks for biophysical parameters (e.g., matrix stiffness, perfusion rates, oxygen gradients), the incorporation of modular biointerfaces for vascular and immune integration, and the design of closed‐loop systems that enable real‐time monitoring of electrophysiological or metabolic responses. Perfusable bioreactors, advanced hydrogel scaffolds, and microfluidic compartmentalization will be essential to support long‐term culture, dynamic signaling, and therapeutic screening. *OrganoidMap* can support this evolution by integrating data on material interfaces, biomechanical features, and hybridization strategies, enabling systematic comparison across next‐generation platforms. In doing so, it offers a framework for aligning cerebral organoid research with the broader trajectory of engineered living systems, enhancing reproducibility, interoperability, and translational potential at the interface of biology and bioengineering.

### 3.2 From Landscape‐Level Overview to Targeted Discovery

While this review does not provide a detailed analysis of individual studies or highlight specific models in depth, this was a deliberate choice aligned with our aim to offer a comprehensive, landscape‐level synthesis. Given the scope of 738 original studies, the objective of the text is to chart the overall structure of the cerebral organoid literature, spanning domains, methodologies, and model characteristics, rather than to focus on individual applications.

In contrast, *OrganoidMap* enables detailed exploration of all studies included, allowing users to filter and identify subsets aligned with their specific interests. Together, the review and platform serve as a starting point for more focused, disease‐specific, or technique‐driven reviews, as well as comparative investigations across model systems.

### 3.3 Cerebral Organoids: Opportunities and Constraints

Cerebral organoids offer distinct advantages over conventional systems. Their self‐organizing architecture enables modeling of spatial organization, lineage specification, and intercellular communication in ways not possible in static cultures. The use of patient‐derived iPSCs allows for personalized modeling, while advances in gene editing and imaging technologies enable high‐resolution exploration of disease mechanisms. As summarized in our review, organoids have proven powerful for modeling neurodevelopmental disorders, genetic tauopathies, and glioma heterogeneity, with growing promise in therapeutic response profiling. However, significant constraints remain. Most systems retain fetal‐like maturity, limiting their utility for modeling late‐onset neurodegenerative diseases or capturing aging‐associated trajectories. Key physiological components, including vasculature, immune niches, and cerebrospinal interfaces, are typically absent or incompletely integrated. Batch variability, lack of protocol harmonization, and inconsistent functional readouts continue to affect reproducibility and scalability. Disease modeling is also biased, with a focus on glioblastoma, tauopathies, and autism spectrum conditions, while lower‐grade gliomas, cerebrovascular insults, and many neuroimmune disorders remain understudied. Crucially, few studies systematically validate organoid phenotypes against clinical data or therapeutic outcomes. Without longitudinal benchmarking or prospective clinical correlation, the translational value of these systems remains largely inferential. To advance toward clinically actionable tools, organoids must evolve not only in biological complexity but also in validation pipelines, standardization efforts, and integration into clinically relevant frameworks.

### 3.4 Organoids in Functional Precision Medicine

To fulfill their translational promise, organoid systems must evolve from hypothesis‐generating platforms into functionally predictive tools. While genomic stratification is increasingly standard in oncology, functional readouts, particularly those assessing both therapeutic efficacy and neurotoxicity, remain underdeveloped. This gap is critical: in clinical practice, therapeutic failure often results from unexpected toxicity or non‐response, outcomes that are rarely captured by static genomic profiles alone. Organoids offer a complementary strategy, enabling individualized models that reflect patient‐specific disease mechanisms, drug sensitivity, and adverse effect susceptibility. To be clinically actionable, however, these models must achieve standardization, scalability, and temporal maturity. This includes the capacity to support longitudinal assays, define therapeutic windows, and map functional responses across both diseased and healthy neural tissues. By bridging the gap between molecular profiles and functional outcomes, organoids could help refine therapeutic windows and guide more informed, individualized treatment decisions across both neurological and oncological conditions. However, prospective clinical trials integrating organoid phenotyping remain rare. Yet, the increasing availability of patient‐derived tissue provides a powerful, but underutilized, opportunity for individualized disease modeling. However, meaningful clinical translation will require integrated workflows, ethical frameworks, and interdisciplinary collaboration. Organoids can only deliver on their promise if embedded within the clinical ecosystem—supporting real‐time, functionally informed patient stratification. Ultimately, the make‐or‐break point for organoids in medicine may not be technical feasibility, but whether they are embraced as a bridge between experimental insight and clinical decision‐making.

### 3.5 Species‐Model Divergence and the Need for Rodent Organoids

Our literature review revealed that over 95% of studies utilized human‐derived cells or tissue, underscoring a strong and growing preference for human‐specific systems. While this reflects the ethical and translational appeal of organoid platforms, it also reveals a less obvious but critical limitation: the near‐complete absence of complementary rodent‐derived organoids. This gap presents a challenge in preclinical workflows, where regulatory agencies (e.g., FDA, EMA) continue to require rodent *in vivo* validation, yet findings from human organoids and murine models often diverge. In such cases, it remains unclear whether discrepancies arise from species‐specific biology or from differences in model architecture (e.g., *ex vivo* vs. *in vivo*). Rodent organoids could serve as an intermediate system, retaining tissue complexity while enabling scalable, ethically compliant, and directly comparable preclinical analyses. As complementary platforms, they would allow parallel testing across species and models, helping to resolve model‐versus‐species effects, reduce translational noise, and accelerate therapeutic development.

### 3.6 Ethical and Regulatory Considerations

As cerebral organoid technologies advance, ethical and governance considerations are becoming increasingly salient and can no longer be treated as purely anticipatory. Although current cerebral organoids do not meet established criteria for sentience or consciousness, ongoing efforts to enhance longevity, interregional connectivity, and functional maturation are progressively narrowing the conceptual gap between in vitro neural models and *in vivo* systems.

Importantly, ethical complexity is not limited to future scenarios. Several studies have already demonstrated the successful transplantation of cerebral organoids into murine brains, where grafted tissue survives, vascularizes via host integration, and undergoes progressive maturation, including synaptic integration and functional coupling with host circuits. These chimeric models underscore that organoids can participate in living neural environments, blurring traditional boundaries between in vitro models and animal experimentation. While such studies remain scientifically valuable and ethically permissible under existing frameworks, they make clear that cerebral organoids are not static culture systems and that their moral and regulatory status may evolve as functional integration increases.

In parallel, the expanding use of patient‐derived iPSCs introduces additional ethical challenges related to informed consent, long‐term biobanking, and data governance. Many consent frameworks were developed for fixed experimental endpoints and do not explicitly address open‐ended organoid use, longitudinal functional profiling, or the possibility that organoid‐derived data may inform clinical decision‐making. As organoids increasingly serve as platforms for therapeutic screening or personalized modeling, questions surrounding data ownership, return of results, and clinical responsibility become increasingly relevant.

Regulatory oversight further remains fragmented. Existing ethical guidelines are largely adapted from stem cell research or animal experimentation and do not fully capture the hybrid nature of cerebral organoids, particularly in contexts involving chimeric transplantation, long‐term culture, or advanced functional readouts. International variation in governance frameworks compounds this challenge and highlights the need for coordinated, field‐specific standards.

To ensure responsible and socially aligned progress, the cerebral organoid community should proactively engage with these issues by: (1) Developing organoid‐specific consent and governance frameworks, particularly for patient‐derived and clinically linked models; (2) Establishing clear ethical guidelines for chimeric transplantation studies, including criteria for monitoring functional integration; (3) Promoting transparency and standardization in reporting model complexity, maturity, and downstream applications; and (4) Integrating ethics expertise into translational organoid research pipelines from early experimental stages.

Addressing these considerations proactively will be essential to maintaining public trust and ensuring that cerebral organoid research advances within ethically robust, scientifically rigorous, and socially responsible boundaries.

## 4 Limitations and Future Directions

This review provides the first cross‐domain synthesis of cerebral organoid research, yet several limitations must be acknowledged. First, the search was limited to peer‐reviewed, English‐language publications indexed in PubMed, Semantic Scholar, and OpenAlex, which may have excluded relevant non‐English studies, preprints, or gray literature. Second, the PubMed query used singular keyword terms (“cerebral” AND “organoid”) without truncation or controlled vocabulary (e.g., MeSH terms). This design choice prioritized specificity and reduced false positives but may have excluded studies using alternative terminology (e.g., “neural organoids,” “brain spheroids”) or those not accurately indexed. To mitigate this, full‐text searches were expanded via Semantic Scholar and OpenAlex. Third, this review was not preregistered in PROSPERO or a similar registry. This decision reflects the exploratory and integrative nature of the project, which evolved alongside the development of *OrganoidMap*, requiring iterative design decisions. We acknowledge the value of preregistration and intend to incorporate it into future updates. Fourth, a meta‐analysis was not conducted, due to extensive heterogeneity in experimental designs, model systems, cell sources, and outcome measures. The synthesis is therefore narrative and descriptive, intended to map the structure and gaps of the literature rather than quantify effect sizes. Fifth, despite employing a structured classification system, data extraction inevitably abstracts experimental nuance, particularly in studies lacking standardized terminology or reporting reproducibility indicators. Relatedly, many primary studies omit negative results, functional readouts, or replication data, limiting the ability to assess model robustness systematically. As a result, the quality and depth of available evidence remain uneven across domains. Sixth, although we identified 738 original studies, the field is evolving rapidly, and indexing inconsistencies may have led to unintentional omissions. The current dataset reflects literature up to the extraction date of October 9, 2024, and should be interpreted as a temporally bounded snapshot. Seventh, the decision to prioritize broad coverage across neurodevelopment, neurodegeneration, and neuro‐oncology necessarily limited the interpretive depth within any single disease category. However, this design enables structured comparison across domains, a perspective often absent in disease‐focused reviews, and provides a foundation for more specialized downstream analyses. Finally, while *OrganoidMap* is unique in scope, it remains an initial iteration. The current version enables filtering by key parameters such as disease context, model type, and methodological features. Although static for this publication, the platform will be updated regularly to incorporate new literature, community feedback, and functional enhancements. These updates will expand its utility not only as a reference database, but also as a dynamic tool for model comparison, experimental design, and cross‐domain integration. As organoid complexity increases and translational goals intensify, continuous refinement of both data and infrastructure will be critical to ensuring reproducibility, interoperability, and clinical relevance.

Beyond serving as a comprehensive reference, *OrganoidMap* is intended as a conceptual framework for the next generation of cerebral organoid research. By structuring models according to biological scope, technical complexity, and application domain, the platform enables comparative benchmarking across protocols and highlights design gaps relevant to vascularization, immune integration, and bioengineering interfaces. In this way, *OrganoidMap* can guide the rational development of hybrid systems, such as organoid‐on‐chip platforms, perfused bioreactors, and multicellular assembloids, by making existing trade‐offs in fidelity, scalability, and functionality explicit. As the field moves toward increasingly complex and translationally oriented models, such integrative tools will be essential not only for navigating the literature, but for shaping future experimental strategies.

## 5 Conclusion

This work presents the first comprehensive, cross‐domain synthesis of cerebral organoid research, mapping 738 original studies across neurodevelopment, neurodegeneration, and neuro‐oncology. To translate this synthesis into a usable resource, we developed *OrganoidMap*—an open‐access, interactive platform that enables structured exploration and comparison of models at scale. By providing searchable access to curated information on model types, disease applications, and methodological features, *OrganoidMap* transforms a fragmented field into a navigable, comparative research landscape. In doing so, it addresses key challenges in reproducibility and transparency, while setting a new benchmark for open, data‐integrated scientific infrastructure. This integration of systematic review and interactive dissemination converts static synthesis into a living resource, empowering researchers not only in neuroscience but across bioengineering, materials science, and translational medicine to make more informed decisions in model selection, benchmarking, and experimental design. As the complexity and utility of organoids continue to grow, tools like *OrganoidMap* will be essential to advancing innovation at the interface of biology, engineered systems, and clinical application.

## 6 Methods

### 6.1 Systematic Review Design

This systematic review aimed to map and synthesize the use of 3D *ex vivo* cerebral organoid models in neuroscience research, specifically across the domains of neurodevelopment, neurodegeneration, and neuro‐oncology. Articles published between January 1, 2014, and October 9, 2024, were included. The review followed the PRISMA 2020 guidelines and was designed to integrate insights from a fragmented and rapidly evolving field into a unified, accessible resource.

### 6.2 Search Strategy

A systematic literature search was conducted across three databases: PubMed, Semantic Scholar, and OpenAlex. The search was limited to English‐language articles published between January 1, 2014, and October 9, 2024. This timeframe reflects the emergence and rapid adoption of cerebral organoid technologies in neuroscience.

In PubMed, we used the Advanced Search Builder with the keyword‐based query: “cerebral” AND “organoid”. This query used singular terms only, a deliberate choice based on preliminary benchmarking. PubMed's automatic term mapping typically retrieves plural forms (e.g., “organoids”) when the singular is entered. Broader truncation (e.g., organoid*) significantly increased the number of off‐topic results, particularly studies unrelated to cerebral organoids. To preserve relevance while maintaining manageable screening volume, we opted for a specific and precise search formulation. We did not use controlled vocabulary (e.g., MeSH terms), as many recent and relevant studies were not consistently indexed under such headings. To improve coverage and minimize indexing bias, we supplemented the PubMed search with keyword‐based full‐text searches in Semantic Scholar and OpenAlex, which are not constrained by indexing fields and offer broader access to interdisciplinary literature. Preprints and non‐peer‐reviewed materials were excluded across all platforms.

### 6.3 Inclusion and Exclusion Criteria

Articles were eligible for inclusion if they were original research using cerebral organoids or closely related 3D brain models in the context of neurodevelopment, neurodegeneration, or neuro‐oncology. The selection process included the following stages: (1) Database Export and Deduplication: Search results were exported in .xls format from each database and merged into a single dataset. Duplicates were removed using Excel's “highlight duplicates” function, followed by manual verification to ensure accuracy; (2) Preliminary Screening: Titles and abstracts were reviewed to exclude: non‐original research (e.g., reviews, commentaries, editorials); non‐peer‐reviewed materials (e.g., preprints, theses, conference abstracts); articles without accessible full text; and (3) Full‐Text Screening: Despite prior filtering, some misclassified or non‐original articles were identified during full‐text review, often due to inconsistent metadata across databases. These were excluded accordingly. Final exclusions were categorized as: R1: Studies not involving a 3D brain model; R2: Off‐topic studies (e.g., non‐cerebral organoids, computational‐only models); and R3: Non‐original or non‐peer‐reviewed research missed during earlier screening.

Screening was conducted independently by five authors (A.W., A.M., V.A., M.S., and M.V.), with disagreements resolved through discussion.

### 6.4 Data Extraction

A standardized Excel template was used to extract structured data from each included article. During full‐text review, articles were classified by: Domain (neurodevelopment, neurodegeneration, neuro‐oncology); Disease subtype; Model type (e.g., cerebral organoid, assembloid, co‐culture); Cell source (e.g., iPSCs, ESCs, NSCs); Application, along with any explicitly stated or implicit advantages and limitations. Data extraction was performed independently by multiple authors. Discrepancies were resolved by consensus.

### 6.5 Dataset Finalization and Public Accessibility

Data extraction was completed on October 9, 2024. Following this, efforts transitioned toward the development of *OrganoidMap*, an open‐access, interactive platform designed to present, filter, and explore the curated dataset. The dataset remains fixed as of the extraction date, ensuring consistency between the publication and the public‐facing resource.

Although the current review reflects the literature up to October 2024, we acknowledge the importance of continued engagement. We plan to publish regular follow‐up reports and dataset expansions to reflect future research developments, user feedback, and enhancements to the *OrganoidMap* platform.

### 6.6 Narrative Synthesis and Protocol Registration

Due to significant heterogeneity in study designs, outcome measures, and reporting standards, a formal meta‐analysis was not feasible. We therefore employed a narrative synthesis approach, aiming to identify patterns, methodological diversity, and underexplored areas across the field.

This review was not preregistered in PROSPERO or a comparable registry. The project originated as an exploratory synthesis aligned with the development of *OrganoidMap*, which required flexibility in scope and data handling. We acknowledge this as a limitation in terms of transparency and reproducibility and intend to preregister future updates or extensions of this work.

### 6.7 Web Application OrganoidMap

The web application *OrganoidMap* (https://OrganoidMap.cs.uni‐tuebingen.de/) was built to enable user‐friendly access, querying, and visualization of the research results of this systematic review. *OrganoidMap* is built with a Flask backend hosting a relational SQLite database of two tables (one abbreviation table, one full research result table) and a vanilla JavaScript‐, CSS‐, and HTML‐based frontend. The application is running within a Docker container hosted at the infrastructure of the Institute for Bioinformatics and Medical Informatics, Tübingen University. A detailed overview of utilized packages and their versions is provided in Table 1.

**TABLE 1:** Resources.

Package	Source
Backend	
*blinker* (version 1.9.0)	https://pypi.org/project/blinker/
*click* (version 8.1.8)	https://pypi.org/project/click/
*Flask* (version 3.1.0)	https://flask.palletsprojects.com/en/stable/
*Flask‐SQLAlchemy* (version 3.1.1)	https://flask‐sqlalchemy.readthedocs.io/en/stable/
*itsdangerous* (version 2.2.0)	https://pypi.org/project/itsdangerous/
*Jinja2* (version 3.1.6)	https://pypi.org/project/Jinja2/
*MarkupSafe* (version 3.0.2)	https://pypi.org/project/MarkupSafe/
*numpy* (version 2.2.4)	https://numpy.org
*pandas* (version 2.2.3)	https://pandas.pydata.org
*python‐dateutil* (version 2.9.0.post0)	https://pypi.org/project/python‐dateutil/
*pytz* (version 2025.2)	https://pypi.org/project/pytz/
*six* (version 1.17.0)	https://pypi.org/project/six/
*SQLAlchemy* (version 2.0.40)	https://www.sqlalchemy.org
*typing‐extensions* (version 4.13.1)	https://pypi.org/project/typing‐extensions/
*tzdata* (version 2025.2)	https://pypi.org/project/tzdata/
*Werkzeug* (version 3.1.3)	https://werkzeug.palletsprojects.com/en/stable/
Frontend	
*Axios* (version 1.8.4)	https://axios‐http.com/
*Unpkg*	https://unpkg.com/
*JsDelivr*	https://www.jsdelivr.com/
*Font Awesome Free* (version 6.7.2)	https://fontawesome.com/
*Tabulator* (version 6.3.0)	https://tabulator.info/
*ECharts* (version 5.6.0)	https://echarts.apache.org/en/

### 6.8 OrganoidMap: Platform Features and Example Workflow

*OrganoidMap* offers three major layers of user feedback to support systematic exploration of organoid‐based research: (1) A flexible keyword search function that operates across all levels of metadata including domains, subgroups, research focus, model types and cell sources; (2) interactive pie charts for rapid, visual interpretation of the global distribution of studies, enabling users to quickly grasp the landscape across multiple dimensions; and (3) a filterable, detailed study table that allows users to explore and refine results based on specific attributes of interest.

**FIGURE 9:**
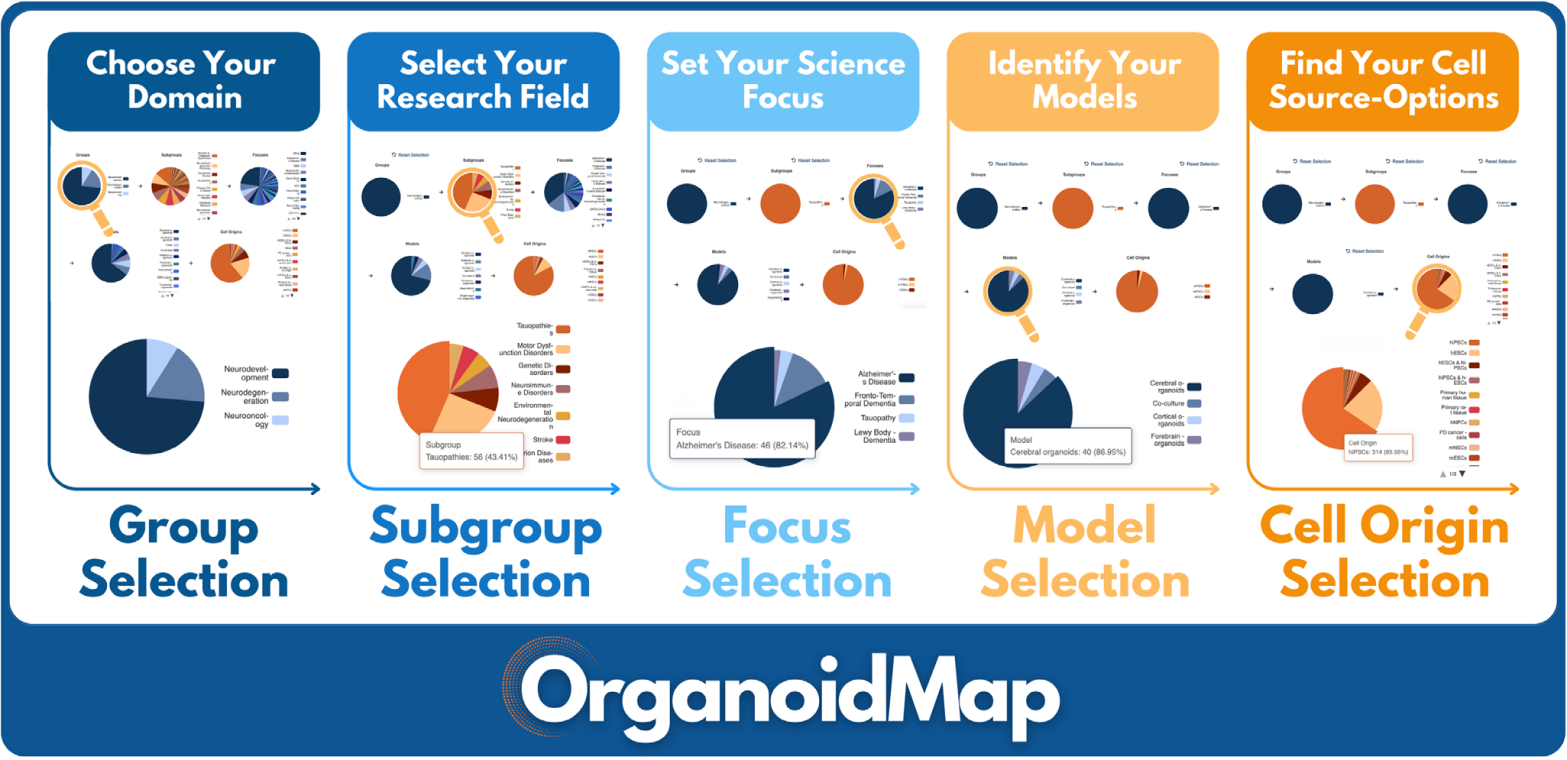
Visual Exploration of Organoid Model Landscape for Alzheimer's Disease. Stepwise pie chart visualization within *OrganoidMap*, demonstrating how users can explore the model landscape for Alzheimer's disease. Beginning with the selection of Group (“Neurodegeneration”), then Subgroup (“Tauopathies”), and finally Focus (“Alzheimer's Disease”), the interface presents interactive pie charts that reveal the distribution of related organoid models (e.g., cerebral organoids) and cell sources (e.g., human iPSCs). This layered, visual approach enables rapid insight into model availability and trends within a specific disease context. Created with Canva (https://www.canva.com).

To illustrate its use, consider a researcher investigating Alzheimer's disease. The user begins by selecting the Group “Neurodegeneration,” then narrows the search to the Subgroup “Tauopathies” and selects “Alzheimer's Disease” as the Focus. The pie chart interface instantly displays aggregated data, highlighting cerebral organoids as the predominant model and human iPSCs as the main cell source used in this context (see Figure 9). The researcher can then access a full list of associated studies via the dynamic table, with options to filter by additional parameters such as publication journal or year.

This example demonstrates how *OrganoidMap* enables fast, intuitive navigation and comparative analysis, supporting informed decision‐making in disease‐specific organoid research.

### 6.9 Data and Code Availability

The *OrganoidMap* web application is available at organoidmap.cs.uni‐tuebingen.de. The corresponding code is available at https://github.com/MaikTungsten/OrganoidMap and archived at https://zenodo.org/records/18420242. All required software is containerized in a Docker image that can be built with all information from the GitHub repository.

## Author Contributions

**Conceptualization**: A.W. (systematic review, web application), M.W.S. (web application), and L.S. **Methodology**: A.W., A.M., V.A., M.S., and M.V. **Software**: M.W.S. (backend implementation) and C.T. (frontend implementation). **Validation**: A.W., V.S.K., and S.A. (proofreading of final database). **Formal Analysis**: A.W., V.A., and A.M. (data analysis and interpretation). **Investigation**: A.M. and M.S. (literature retrieval from PubMed, OpenAlex, Semantic Scholar). **Data Curation**: A.W., A.M., V.A., M.S., and M.V. (study screening and synthesis). **Writing – Original Draft**: A.W. (neurodevelopment in health, neurodegeneration, neuro‐oncology) and V.A. (neurodevelopment in disease). **Writing – Review and Editing**: A.W., V.A., M.W.S., C.R.N., and L.S. **Visualization**: M.W.S. and A.W. (figures and web platform visuals). **Supervision**: C.R.N., A.W., and L.S. (project oversight and critical input). **Project Administration**: A.W. (coordination of design process). **Funding Acquisition**: L.S. (major) and A.W. (minor).

## Funding

This project was funded by institutional funds of the Department of Neurology and Interdisciplinary Neuro‐oncology and the M3 Research Center, Hector Foundation MINT Personal Fonds, the Deutsche Forschungsgemeinschaft (DFG) Cluster of Excellence EXC2180 “Image‐guided and functionally instructed tumor therapy” (iFIT) and Deutsche Forschungsgemeinschaft (DFG) CRC1479 “OncoEscape”– Project ID: INST 39/1479‐1 (awarded to L.S.); and by the Joachim Herz Stiftung (Add‐on Fellowships awarded to V.A. and A.W., and Kickstart Fund awarded to A.W.), as well as the Friedrich‐Ebert‐Stiftung (awarded to A.W.).

## Conflicts of Interest

The authors declare the following conflicts of interest: A European patent application (No. 24 219 517.0) related to cerebral organoids was filed by Tübingen University Hospital on December 12, 2024.

## Declaration of Generative AI and AI‐Assisted Technologies in the Writing Process

During the preparation of this work, the authors used a paid, customized version of ChatGPT (SciWriting) solely to assist with language editing and grammar correction. No content was generated by AI. The authors reviewed and further edited the manuscript and take full responsibility for the content of the published article.

## Supporting information




**Supporting File**: adhm70906‐sup‐0001‐OrganoidMap.xlsx.

## Data Availability

Data extraction for this systematic review was completed on October 9, 2024. The dataset used in this manuscript and presented through the OrganoidMap platform is fixed as of this date to ensure consistency between the publication and the public resource. The full curated dataset is available as an Excel file in the Supplementary Material. The OrganoidMap web application is publicly accessible at https://organoidmap.cs.uni‐tuebingen.de. The source code is hosted on GitHub (https://github.com/MaikTungsten/OrganoidMap) and has been archived on Zenodo (https://zenodo.org/records/18420242)for long‐term preservation. All necessary software dependencies are provided via Docker and can be built using the configuration files available in the GitHub repository. Future updates to the OrganoidMap platform and dataset are planned to incorporate newly published studies, user feedback, and technical improvements.
